# Double-modified, thio and methylene ATP analogue facilitates wound healing in vitro and in vivo

**DOI:** 10.1038/s41598-024-63759-5

**Published:** 2024-06-07

**Authors:** Roza Pawlowska, Ewa Radzikowska-Cieciura, Sepideh Jafari, Julia Fastyn, Eliza Korkus, Edyta Gendaszewska-Darmach, Gangyin Zhao, Ewa Snaar-Jagalska, Arkadiusz Chworos

**Affiliations:** 1 Centre of Molecular and Macromolecular Studies, Polish Academy of Sciences, Sienkiewicza 112, 90-363 Lodz, Poland; 2 BioMedChem Doctoral School of the University of Lodz and the Institutes of the Polish Academy of Sciences in Lodz, Lodz, Poland; 3 Institute of Molecular and Industrial Biotechnology, Faculty of Biotechnology and Food Sciences, Lodz University of Technology, Stefanowskiego 2/22, 90-537 Lodz, Poland; 4 Institute of Biology, Leiden University, 2333 BE Leiden, The Netherlands

**Keywords:** ATP analogue, Wound healing, Skin regeneration, P2Y2 receptor, Purinergic signaling, Biochemistry, Chemical biology

## Abstract

Recent data indicate that extracellular ATP affects wound healing efficacy via P2Y2-dependent signaling pathway. In the current work, we propose double-modified ATP analogue—alpha-thio-beta,gamma-methylene-ATP as a potential therapeutic agent for a skin regeneration. For the better understanding of structure–activity relationship, beside tested ATP analogues, the appropriate single-modified derivatives of target compound, such as alpha-thio-ATP and beta,gamma-methylene-ATP, were also tested in the context of their involvement in the activation of ATP-dependent purinergic signaling pathway via the P2Y2 receptor. The diastereomerically pure alpha-thio-modified-ATP derivatives were obtained using the oxathiaphospholane method as separate *S*_P_ and *R*_P_ diastereomers. Both the single- and double- modified ATP analogues were then tested for their impact on the viability and migration of human keratinocytes. The involvement of P2Y2-dependent purinergic signaling was analyzed in silico by molecular docking of the tested compounds to the P2Y2 receptor and experimentally by studying intracellular calcium mobilization in the human keratinocytes HaCaT. The effects obtained for ATP analogues were compared with the results for ATP as a natural P2Y2 agonist. To confirm the contribution of the P2Y2 receptor to the observed effects, the tests were also performed in the presence of the selective P2Y2 antagonist—AR-C118925XX. The ability of the alpha-thio-beta,gamma-methylene-ATP to influence cell migration was analyzed in vitro on the model HaCaT and MDA-MB-231 cells by wound healing assay and transwell migration test as well as in vivo using zebrafish system. The impact on tissue regeneration was estimated based on the regrowth rate of cut zebrafish tails. The in vitro and in vivo studies have shown that the *S*_P_-alpha-thio-beta,gamma-methylene-ATP analogue promotes regeneration-related processes, making it a suitable agent for enhance wound healing. Performed studies indicated its impact on the cell migration, induction of epithelial–mesenchymal transition and intracellular calcium mobilization. The enhanced regeneration of cut zebrafish tails confirmed the pro-regenerative activity of this ATP analogue. Based on the performed studies, the *S*_P_-alpha-thio-beta,gamma-methylene-ATP is proposed as a potential therapeutic agent for wound healing and skin regeneration treatment.

## Introduction

Non-healing wounds affect a large group of people with chronic diseases, such as various types of cardiovascular disorders, diabetes, and skin-related disturbances, as well as patients after accidents or surgical procedures^1,2^. This problem affects nearly 2.5% of the total population of United States^1^ and the number of patients with chronic wounds among the Medicare beneficiaries is still growing. The increase between 2014 and 2019 was from 14.5 to 16.3%, respectively^3^. In the Europe, it is estimated that 1,5—2 million people are struggling with this problem^4^. This data clearly demonstrates the importance of working on new strategies to enhance the wound healing.

In a proper wound healing process, alterations in skin cells are required to close the wound and regenerate tissue^2,5^. These processes include enhancement of cell migration, proliferation, induction of epithelial–mesenchymal transition (EMT) and collagen production^6,7^. Several factors are involved in proper wound closure^2,5^. The composition of the extracellular microenvironment has been shown to be one of the important factors that can influence tissue regeneration^2,8^, where extracellular nucleotides, especially adenosine-5′-triphosphate (ATP) and uridine-5′-triphosphate (UTP), are key elements, involved in these processes^9,10^.

Adenosine triphosphate is a well characterized multifunctional molecule, which functions as an energy storage, substrate and cofactor of several enzymes and signaling molecule^11^. In the extracellular environment ATP and its cognate receptors, both G protein-coupled P2Y and ligand-gated ion channel P2X receptors, represent autocrine and paracrine regulation systems, which are widespread inside the body and may regulate numerous processes associated with the proper functioning of the main tissues, organs and whole systems^11–13^. The extracellular ATP-dependent signaling is crucial for regulation of cell proliferation, differentiation and death occurring during development and regeneration processes^11^. The autocrine and paracrine adenine nucleotides based communication occurred to play important role in immune cells^13^. Interestingly, a similar phenomenon has been observed also in cancer cells^14^. Some disturbances in ATP-based communication system have been observed in various pathological states in urinogenital, airway, musculoskeletal and gastrointestinal systems, immune pathogenesis, cardiovascular pathophysiology^11,15,16^.

Due to the widespread presence of nucleotide-based communication and its involvement in the regulation of many key pathways, therapeutic utilities of purinergic signaling have been considered for years^15,17,18^. However, it is known that unmodified nucleotides, present in the extracellular environment, are rapidly degraded by numerous enzymes^19–23^. To avoid this, various nucleotide analogues with modifications at different sites, both in the ribose part, nucleobase and within the phosphate group have been designed and obtained to date^18^. In current research, we have focused on the phosphate-modified ATP analogues as more stable ATP counterparts. The introduction of modifications into the phosphate part of nucleotide usually provides partial protection against the hydrolyzing enzymes^18,23^. The interesting representatives of nucleotides with prolonged lifetimes are molecules containing methylene modification. The methylene analogues of ATP: adenylyl 5′-(alpha,beta-methylene)-diphosphonate (α,β-methylene-ATP) and adenylyl 5′-(beta,gamma-methylene)-diphosphonate (β,γ-methylene-ATP), have been shown to interact with P2X purinergic receptors and due to their higher resistance to nucleolytic degradation have been presented as stable P2X receptors agonists^24^. The β,γ-methylene-ATP was recently shown to inhibit vascular smooth muscle cell calcification in vitro and to affect bone mineralization and arterial media calcification in rats^25^. Thus, the potential role of β,γ-methylene-based nucleotide analogues in preventing arterial calcifications has been considered^25^.

Methylene analogues of ATP are not only referred as stable compounds, but also have been proposed as promising inhibitors of nucleotide pyrophosphatase/phosphodiesterase-1 (NPP1), one of the enzymes, which is involved in hydrolysis of extracellular nucleotides^26^. Although, both methylene-modified ATP analogues (α,β-methylene-ATP and β,γ-methylene-ATP) are referred as non-hydrolysable, they are known to be partially degraded^23,27^. Furthermore, the α,β-methylene-ATP may act as a substrate for ecto-nucleotide diphosphokinases (NDPKs) and may be used for trans-phosphorylation of ADP, which has been demonstrated with both purified enzyme and under cell culture conditions^28^. The β,γ-methylene-ATP derivative exhibits higher stability, because the introduction of modification between β and γ phosphorus atoms prevents the exchange of the γ-phosphate between this molecule and other nucleoside diphosphates (NDP). However, a conversion of β,γ-methylene-ATP to AMP and adenosine is still present^27^. To obtain more stable counterparts, additional modifications were needed. The example of the double modified ATP analogue containing with enhance stability is 2-hexylthio-β,γ-methylene-ATP, which was shown to be stable to hydrolysis by nucleoside triphosphate diphosphohydrolases (NTPDase) 1, -2, -3, and -8^29^.

A common way to achieve resistance to enzymatic degradation is the introduction of a sulfur atom in place of one of the non-bridging oxygen in the phosphate part of the nucleotide^23,30^. Phosphorothioate modified nucleotides are presented as a good synthetic tool for molecular biology purposes^31,32^. Introduction of thio-modification into oligonucleotides is known to provide increase of hydrolytic stability and influence activity of such changed molecules^30^. Interestingly, phosphorothioate modification is not only a product of chemical synthesis, but also has been discovered in naturally occurring nucleic acids^30^, which makes it particularly interesting.

Thio-modified ATP analogues, as metabolically more stable compounds than ATP, are commonly used for the analysis of ATP-dependent signaling pathways. The introduction of a phosphorothioate creates a new chiral centre, which results in obtaining two diastereomers of each α-thio modified compound. Despite the fact, that the pairs of isomers differ only in a spatial configuration at the phosphorus centre, they may reveal different biological activities. The differences in the interaction of particular P-diastereomers with various natural targets have been presented for certain α-thio-modified nucleotide analogues in both theoretical analyses^33^ and experimental studies^26,31^. Such data indicate the necessity for analysis of individual compounds for each pair of diastereomers specially in the view of interaction with specific receptors.

**Figure 1:**
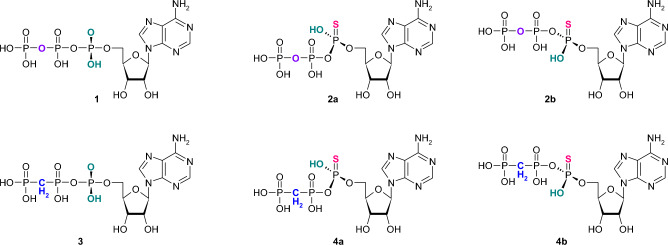
ATP derivatives used in current studies: ATP (**1**), α-thio-ATP (**2a,b**), β,γ-methylene-ATP (**3**) and α-thio-β,γ-methylene-ATP (**4a,b**), respectively.

Literature data have shown that extracellular nucleotides may enhance wound healing via interaction with membranous purinergic receptors and thus induction of purinergic signaling^9,10,34,35^. Based on these reports, we have investigated the potential utilities of double modified ATP analogue—α-thio-β,γ-methylene-ATP, as well as its single-modified counterparts, like α-thio-ATP and β,γ-methylene-ATP (Fig. 1), in the support of skin regeneration processes. For this purpose, ATP derivatives were synthesized and separated to pure *S*_P_ and *R*_P_ diastereomers and then their involvement in induction of ATP-dependent purinergic signaling accompanied to wound healing have been tested. Processes related to tissue regeneration have been examined in vitro and in vivo.

## Results

### The individual diastereomers of α-thio-β,γ-methylene-ATP and α-thio-ATP have been obtained and tested for their cytotoxicity and impact on migration properties of human keratinocytes

#### The α-thio-ATP and α-thio-β,γ-methylene-ATP analogues have been synthesized and separated into individual diastereomers

**Figure 2:**
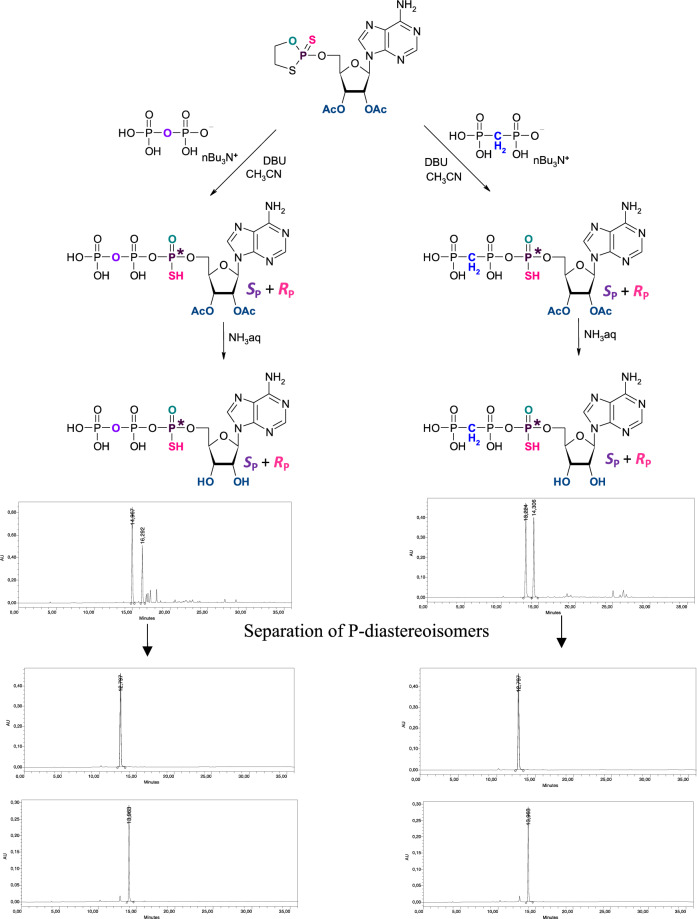
The schematic procedure for synthesis and purification of diastereomerically pure α-thio-modified ATP analogues. The single-modified **2a** and **2b** derivatives are presented in the left panel, double-modified **4a** and **4b** compounds in the right panel. The general synthesis scheme (upper part) and HPLC profiles below including: post-synthetic mixture of P-diastereoisomers **2a** and **2b**, **4a** and **4b**, respectively (top), followed by profiles after separation of compound **2** and **4** into individual P-isomers: fast (middle) and slow (lower), respectively, according to their chromatographic mobility on a C18 column.

The double-modified ATP analogue—adenosine 5′-*O*-(P-α-thio-β-γ-methylenetriphosphate) **(α-thio-β,γ-methylene-ATP**, **4**), as well as its single-modified derivatives: adenosine 5′-O-(P-α-thio-triphosphate) (**α-thio-ATP**, **2**) and adenosine 5′-*O*-(P-β-γ-methylenetriphosphate) **(β,γ-methylene-ATP**, **3**) were designed to be analyzed for their possible interaction with the P2Y2 receptor and thus for their involvement in the induction of purinergic signaling for skin and tissue regeneration. Five compounds **1**, **2a**, **2b**, **3**, **4a** and **4b** (Fig. 1) have been analyzed in the context of their impact on human keratinocytes. Compounds without thio-modification, i.e. **1** and **3** have been purchased, whereas all of the α-thio-modified ATP analogues, both: double modified α-thio-β,γ-methylene-ATP, (**4**) as well as single modified α-thio-ATP (**2**) have been synthesized in the form of diastereomerically pure compounds referred as **2a**, **2b** and **4a**, **4b**, respectively. The syntheses were performed using the oxathiaphospholane (OTP) method^36^. The method was originally developed by Stec for the stereocontrolled synthesis of P-chiral phosphorothioate analogues of oligodeoxyribonucleotides^30^. The application of the OTP method for thiophosphorylation and phosphorylation of various alcohols, as well as, for the synthesis of different nucleoside di- and triphosphates has also been presented^37,38^. The approach is based on the usage of nucleoside-(2-thio-1,3,2-oxathiaphospholane) derivatives as key intermediates. The appropriately protected adenosine 5′-*O*-(2-thio-1,3,2-oxathiaphospholane) was obtained, according to the described procedure, by the phosphorylation reaction of the 5′-hydroxyl group of 2′,3′-di-*O*-acetyladenosine with 2-chloro-1,3,2-oxathiaphospholane in the presence of elemental sulfur. The next step is the reaction with equimolar amounts of pyrophosphate, as described previously^39^, or methylene diphosphonate anion in the presence of DBU as a base catalyst (Fig. 2). To exclude moisture, the reaction was carried out in anhydrous acetonitrile (stored over 3Å molecular sieves) and the substrates used in the reaction were dried together under high vacuum overnight before use (for details see M&M part). The ring opening reaction followed by spontaneous elimination of ethylene sulfide has led to the expected compounds **2** and **4**. The protecting acetyl groups were then removed by ammonia treatment at room temperature for 2 h and α-thio-ATP (**2**) and α-thio-β,γ-methylene-ATP (**4**) were subsequently isolated from the reaction mixture by means of ion exchange chromatography on DEAE-Sephadex A25 in 55% and 47% yields in a form of P-diastereomeric mixtures, respectively. Each of the α-modified compounds was obtained in the form of the pair of P-diastereomers. Then, the individual diastereoisomers of α-thio-β,γ-methylene-ATP (**4a** and **4b**) and α-thio-ATP (**2a** and **2b**) have been separated using reverse phase high performance liquid chromatography (RP-HPLC) with linear gradient 0–30% MeCN supplemented with 0.1 mol/L TEAB buffer (pH 7.5) as shown in Fig. 2. After the separation, the purity of products was analyzed using analytical HPLC and ^31^P NMR spectroscopy (detailed data are presented in Supplementary Materials). The final quality of compounds was confirmed by analytical RP-HPLC analysis (Fig. 2 and Supplementary Materials).

The configuration of the phosphorus atom in individual compounds was assigned based on the previous studies, which showed that retention time of thio-modified nucleotides during RP-HPLC analysis correlates with an absolute configuration of these P-diastereomers, where the P-diastereomers of the *S*_P_ configuration exhibit shorter retention time compared to their *R*_P_ counterparts^40,41^. These data indicated that the fast migrating isomers **2a** and **4a** have *S*_P_ configuration, whereas their **2b** and **4b** counterparts are *R*_P_ isomers. The assignment based on the mobility of individual P-diastereomers during RP-HPLC analysis corroborates with ^1^H NMR spectroscopy data, which revealed the difference in the chemical shifts of H8 proton between the P-diastereomers (Table S1). According to the previous computational studies^26,42^ in case of the *S*_P_ isomer the H8 signal should be more shielded than for *R*_P_ isomer as a result of interaction with negatively charged alpha phosphorous moiety. Interestingly, in our studies the observed δ value of H8 shift in ^1^H NMR for fast and slow isomers of α-thio-β,γ-methylene-ATP was 8.56 vs 8.49 ppm, respectively. Moreover, in case of compound **2** the H8 signal of isomer fast was also shifted to a lower field in comparison to isomer slow as it is presented in Table S1 (8.48 *vs*. 8.42). Based on both, the chromatographic mobility and chemical shift of H8 proton in NMR analysis, the absolute configuration at alpha phosphorus atom of α-thio-modified-ATP derivatives has been assigned as *S*_P_ for isomers **2a** and **4a** and *R*_P_ for isomers **2b** and **4b**, respectively.

#### The ATP derivatives were not toxic for human keratinocytes, though the differences for S_P_ and R_P_ diastereomers were detected

**Figure 3:**
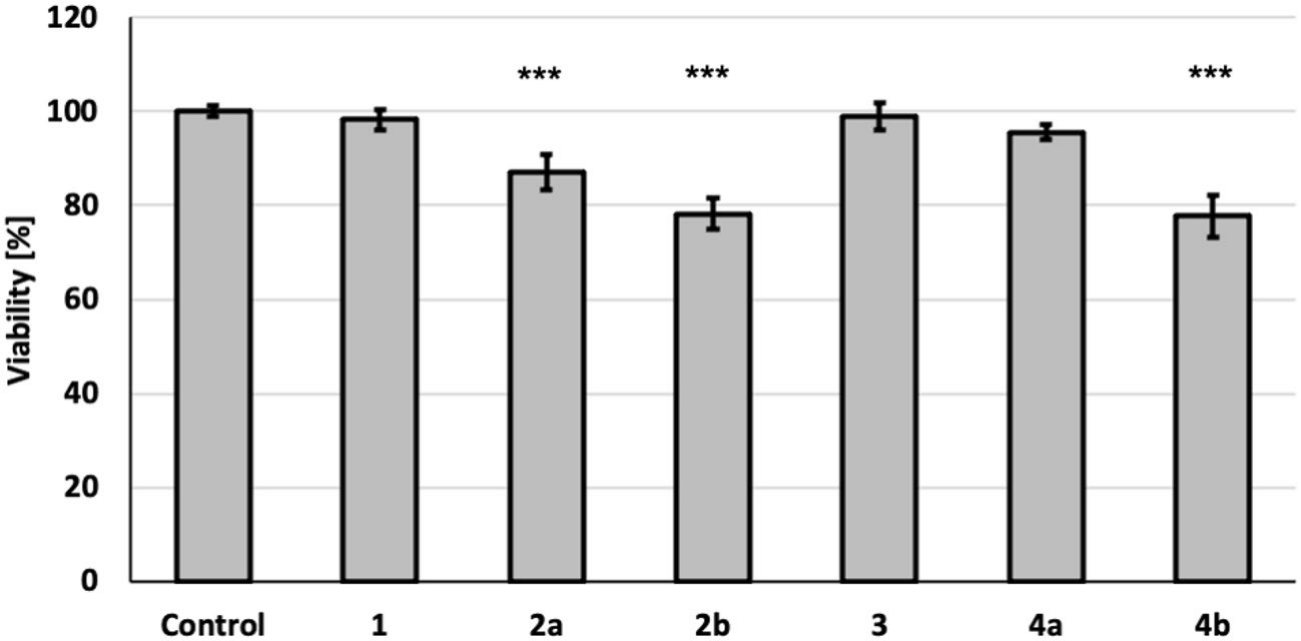
The viability of HaCaT cells after 72 h incubation with tested compounds at the concentration of 100 μM. Data represent mean percentage viability ± standard error of measurement (SEM) estimated for the control (untreated cells, assumed as 100%) from at least 3 independent experiments performed in triplicate. ***p < 0.0001 compared to the control.

Since the ATP analogues have been targeted to skin regeneration, their influence on the viability of skin cells had to be examined. Obtained results confirmed the non-toxic nature of tested compounds for human keratinocytes HaCaT. The maximum value of cytotoxicity after 72 h incubation of cells with tested analogues at concentration of 100 μM did not exceed 20% (Fig. 3). Interestingly, the influence of *R*_P_ diastereomer was stronger than for *S*_P_ in both pairs of α-thio-modified ATP derivatives. The observed differences between *S*_P_ and *R*_P_ diastereomers in both cases may indicate a different specificity of interaction with the target, which determines the activity of tested compounds.

A slight decrease in keratinocytes viability after treatment with single modified α-thio-ATP (**2**) was observed previously^34^ however it was tested only for the mixture of diastereomers. In the current work, skin cell viability after treatment with separated diastereomers of α-thio-ATP and α-thio-β,γ-methylene-ATP derivatives is presented for the first time. The analysis of HaCaT cells viability after treatment with **2a** and **2b** compared to the control (untreated) cells in a timepoint 0, 12, 24, 48 and 72 h were also performed in the assay based on the crystal violet staining to confirm safety of potential use in HaCaT cells (Fig. S12).

For α-thio-β,γ-methylene-ATP analogues (**4a** and **4b**) cytotoxicity also depended on the structure of individual diastereomer. In this case, a reduction of cell viability was also observed for *R*_P_ diastereomer. Interestingly, the cellular viability result obtained for diastereomer **4b** was similar as for the same diastereomer of α-thio analogue without methylene modification (**2b**), while compounds **1**, **3** and diastereomers** 4a** did not significantly affect cell survival after 72-h incubation (Fig. 3). Based on our knowledge, the effect of α-thio-β,γ-methylene-ATP derivatives on skin cells has also never been investigated. Obtained results have indicated that among tested compounds the *S*_P_-α-thio-β,γ-methylene-ATP (**4a**) is free from cytotoxic effects and therefore the most promising ATP analogues for skin applications.

### Impact of diastereomerically pure ATP analogues on intracellular calcium mobilization, migration of human keratinocytes, EMT-related changes in model cells and in vivo tissue regeneration

#### All synthesized α-thio-modified ATP derivatives induce calcium mobilization in human keratinocytes, while β,γ-methylene-ATP analogue remained inactive

**Figure 4:**
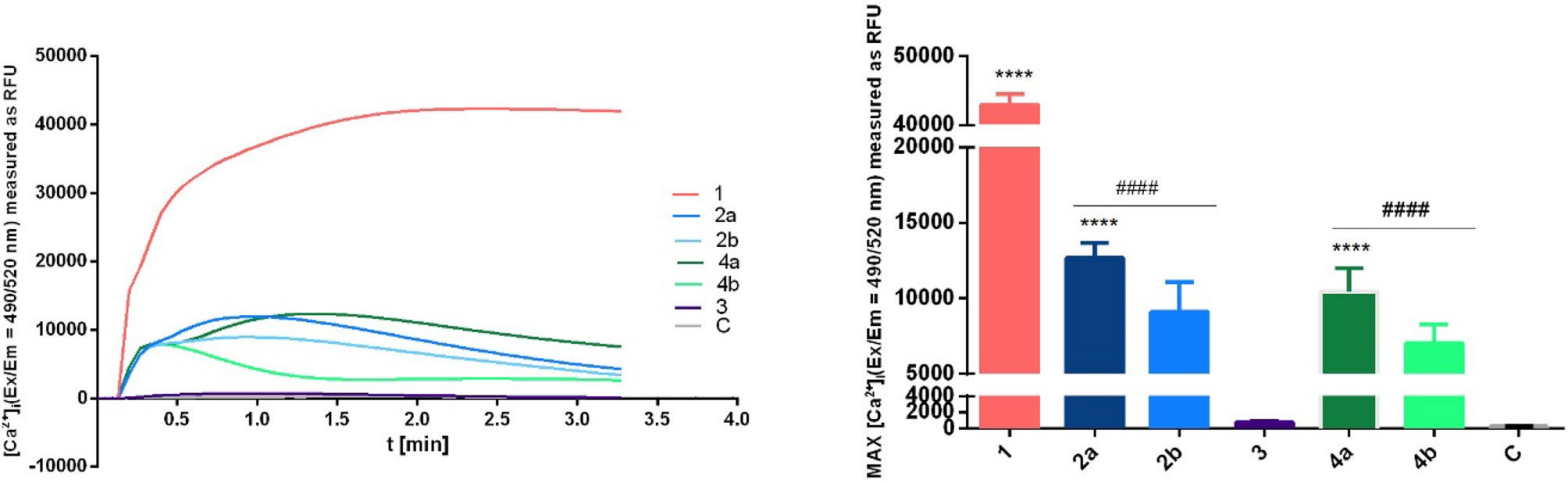
Impact of diastereomerically pure ATP analogues on intracellular calcium mobilization. Intracellular calcium mobilization measurements. The following designations were adopted for compounds: ATP (orange) and tested ATP analogues: α-thio-ATP (**2a** and **2b** blue), β,γ-methylene-ATP (**3** violet) and α-thio-β,γ-methylene-ATP (**4a** and **4b** green). Data represent the means ± SEM from at least 3 independent experiments. ****p < 0.0001 vs control ^####^p < 0.0001 vs ATP. C—untreated control cells, RFU—relative fluorescence units.

It was demonstrated that extracellular nucleotides may affect wound healing by stimulation of keratinocytes via interaction with ATP-dependent surface receptors. As ATP and numerous ATP derivatives activate purinergic receptors, it was hypothesized, that designed α-thio-β,γ-methylene-ATP derivatives might be active in these pathways. In order to verify the involvement of α-thio- and/or β,γ-methylene-modified ATP analogues in induction of purinergic signaling in human keratinocytes, the changes on intracellular free Ca^2+^ concentration [Ca^2+^]i upon ligands’ treatment was measured. The newly synthesized α-thio-β,γ-methylene-ATP analogues (**4a** and **4b**), as well as α-thio-modified derivatives (**2a** and **2b**), β,γ-methylene-ATP (**3**) and ATP (**1**) were tested for the potential impact on the calcium mobilization with the calcium indicator Fluo-8-AM (Fig. 4).

The performed studies showed that all of the α-thio-modified ATP analogues (**2a**, **2b**, **4a**, and **4b**) elevated [Ca^2+^]i in HaCaT keratinocytes, whereas the β,γ-methylene-ATP (**3**) derivative remained inactive (Fig. 4). The ATP-induced increase in [Ca^2+^]i was shown previously in HaCaT cells^21^, thus obtained result for compound **1** was expected. It can be assumed that the high [Ca^2+^]i value obtained for ATP probably resulted from the activation of all possible ATP-dependent signaling pathways and was the cumulative effect of these interactions, while the results for analogues seem to be more specific.

The lack of significant effect on the calcium mobilization after treatment with β,γ-methylene-ATP (**3**) was observed previously in canine tracheal epithelial cells (TECs)^43^, however due to the different expression of purinergic receptors in different cell types, it had to be verified as well in HaCaT cells. Interestingly, despite the absence of the effect for β,γ-methylene-ATP (**3**), we observed the [Ca^2+^]i increase for both diastereomers of the α-thio-modified β,γ-methylene-ATP derivatives (**4a** and **4b**).

Although the influence on intracellular free Ca^2+^ concentration has been shown for all of the tested α-thio-modified ATP derivatives (**2a**, **2b**, **4a**, **4b**), some differences for individual diastereomers were observed. The α-thio-modified methylene derivatives (**4a** and **4b**) exhibited activity comparable to their counterparts without methylene modification (**2a** and **2b**). In both cases, the *S*_P_ diastereomers (**2a** and **4a**) were more active than their *R*_P_ counterparts (**2b** and **4b**). Both, alteration observed for individual diastereomers combined with the differences in activity of α-thio-β,γ-methylene-ATP derivatives (**4a** and **4b**) and α-thio-unmodified β,γ-methylene-ATP derivative (**3**) prompted us to conduct further studies in this area to explain this phenomenon.

Since in the context of wound healing, the most important function of ATP seems to be its involvement in the induction of purinergic signaling in the wound area, the α-thio-modified derivatives (**2a**, **2b**, **4a**, and **4b**) active in this regard were selected for further studies.

#### The S_P_ diastereomers of the α-thio-ATP and α-thio-β,γ-methylene-ATP support the migration of keratinocytes

The migration of keratinocytes at the injury site is one of the most important elements required for the wound closure. One of the key elements regulating this process are extracellular nucleotides. Studies using self-assembled skin substitutes (SASS) have proven, that nucleotides may enhance skin regeneration processes without altering skin quality^35^.

**Figure 5:**
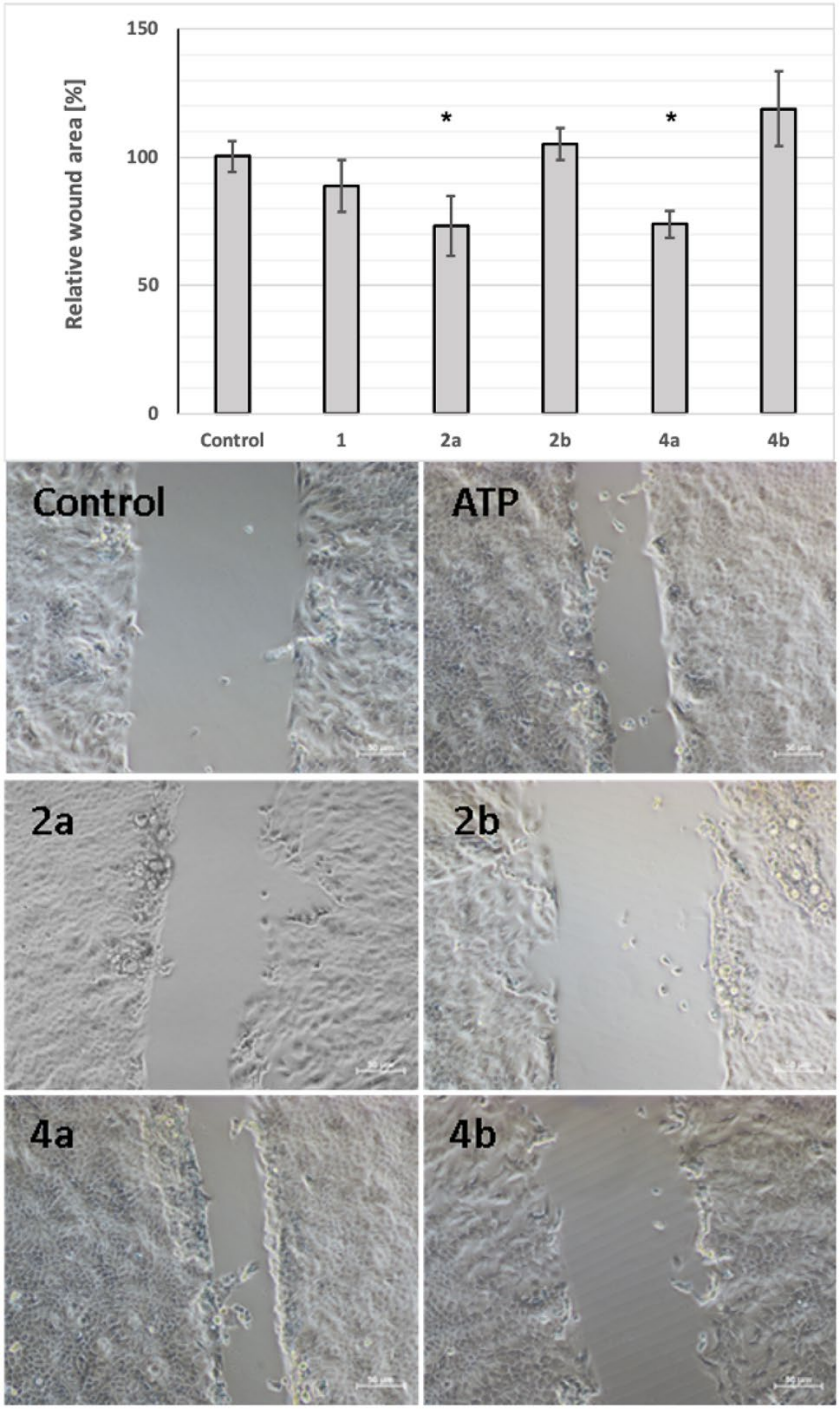
Migration of human keratinocytes after treatment with α-thio-modified ATP analogues. The rate of wound healing after 24 h treatment of HaCaT cells with 100 μM of α-thio-modified ATP derivatives. Upper panel: relative migration rate of HaCaT cells quantified based on the size of the uncovered area after 24 h incubation with tested compounds compared to the untreated cells (taken as control sample 100%). Data represent the means ± SEM from at least 3 independent experiments. Lower panel: the microscopy images of the wound area after 24 h incubation with tested compounds. *p < 0.05 compared to the control, Scale bars 50 μm. The initial sizes of the scratches are available in Supplementary Materials (Fig. S16).

In order to verify, whether studied ATP analogues may affect skin cell migration, HaCaT cell mobility in the presence of tested compounds was assessed using wound healing assay. Obtained results indicated the potential utilities of α-thio-modified ATP analogues in the enhancement of these processes, albeit with some remarks. Performed studies of the scratch assay (Figs. 5, S16) revealed, that in both pairs of analogues, only *S*_P_ diastereomers were active in the promotion of keratinocytes migration (**2a** and **4a**), whereas their *R*_P_ counterparts (**2b** and **4b**), did not support cell migration. After treatment of *S*_P_ diastereomers of α-thio-ATP (**2a**) and α-thio-β,γ-methylene-ATP (**4a**) the size of wound area decreased compared to the control sample (Fig. 5).

#### The α-thio-β,γ-methylene-ATP derivatives induced the EMT-related changes in human model MDA-MB-231 cells

Based on the reports indicated involvement of epithelial–mesenchymal transition (EMT) in wound healing and tissue regeneration^6,44^, it was reasonable to speculate that effect observed after treatment with **4a** and **4b** may be closely tied to the enhancement of the EMT process within cells in the wound surrounding.

**Figure 6:**
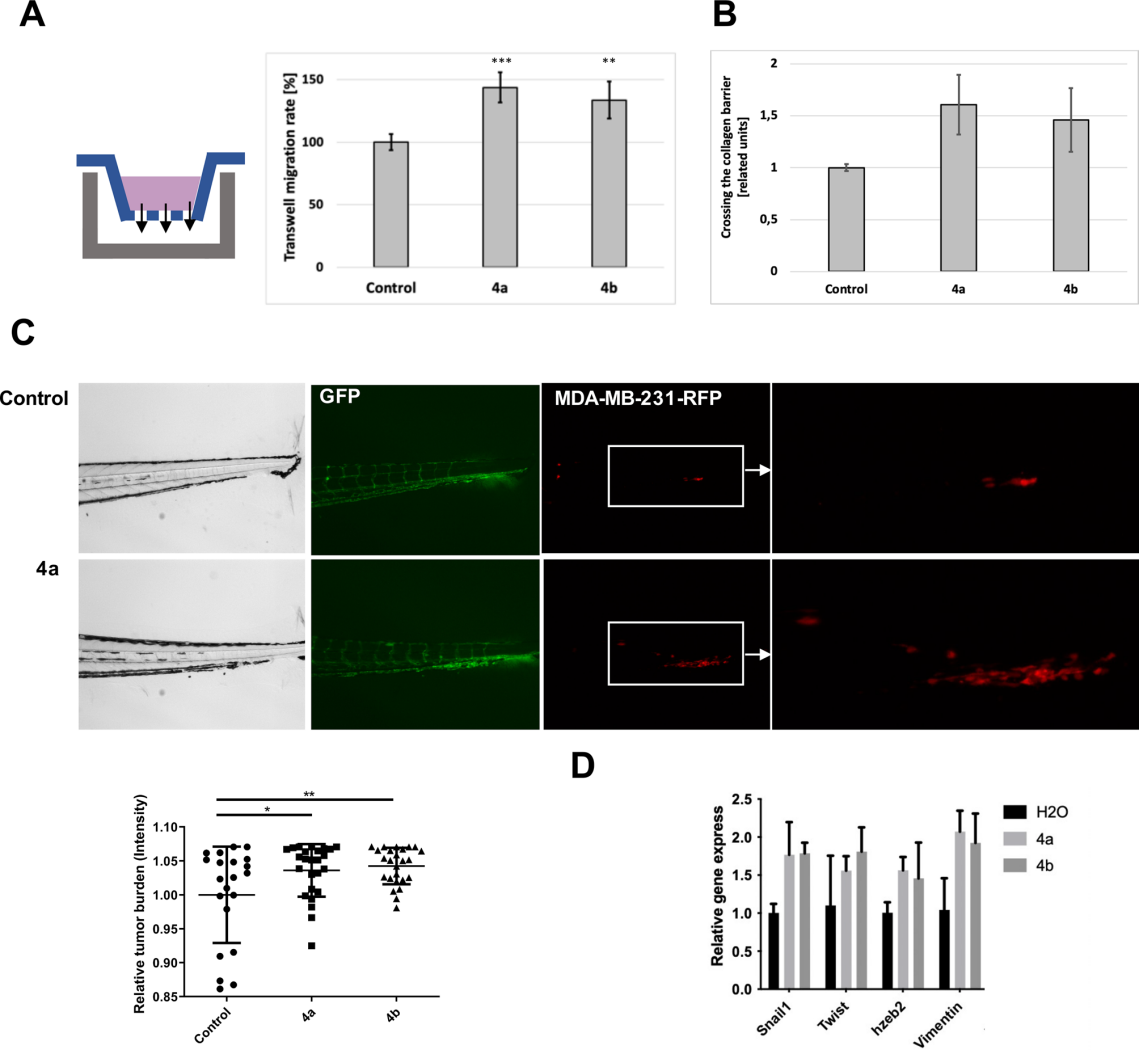
Impact of α-thio-β,γ-methylene-ATP on the epithelial–mesenchymal transition- related changes in the model MDA-MB-231 cells. (**A**) Transwell migration rate of cells through 8 μm pore sizes. Data represent the means ± SEM from at least 3 independent experiments, (**B**) Crossing the collagen barrier in the presence of tested compounds. (**C**) The impact of tested analogues on in vivo cells migration in zebrafish xenograft model (**D**) QPCR analysis of EMT markers after treatment with 4a and 4b. Data represent the means ± SEM from at least 3 independent experiments ***p < 0.0005, **p < 0.001, *p < 0.05 compared to the control.

Firstly, we have checked the effects of α-thio-β,γ-methylene-ATP derivatives on migration rate of cells in 3D conditions, both in vitro—using transwell system and in vivo—in the studies based on the *Danio rerio* xenograft model. The transwell migration analysis revealed the impact of the α-thio-β,γ-methylene-ATP on cells migration (Fig. 6A). It is worth to notice, that values obtained for the **4a** derivative were somewhat better compared to **4b**, however significant increase was observed for both diastereomers. Subsequent studies have shown the impact of **4a** and **4b** on the ability to cross through the polymerized collagen layer (Fig. 6B). The enhancement of 3D cell expansion under the influence of the tested ATP analogues was also observed in vivo in Zebrafish model (Fig. 6C).

Analysis of expression of EMT markers, such as Snail 1, Twist, hzeb2 and vimentin, after treatment of MDA-MB-231 cells with α-thio-β,γ-methylene-ATP derivatives (Fig. 6D) suggested that tested compounds may affect the epithelial–mesenchymal transition in model MDA-MB-231 cells in vitro (Fig. 6D) and in vivo (Fig. S13). The supported immunocytochemical visualization of vimentin in control (untreated) sample and after treatment with compound **4a** is also available in Supplementary Materials (Fig. S17).

Since stimulation of the P2Y2 receptor is known to lead to induction of EMT^45,46^ and facilitates wound healing^8^, it was interesting to verify the potential interaction of α-thio-β,γ-methylene-ATP derivatives with this receptor target.

#### The α-thio-β,γ-methylene-ATP analogues enhance in vivo tissue regeneration after tails cutting in *Danio rerio*

Since all data obtained from in vitro studies on skin cells indicated that designed and synthesized in the current studies double-modified α-thio-β,γ-methylene-ATP may have wound healing promoting activity, it was necessary to check its pro-regenerative potential under in vivo conditions. To check the possible impact of stereoconfiguration on the activity, both diastereomers of synthesized herein α-thio-β,γ-methylene-ATP derivatives (**4a** and **4b**) were used. To verify the activity of compounds in promotion of in vivo tissue regeneration, *Danio rerio* was used as a model organism.

**Figure 7:**
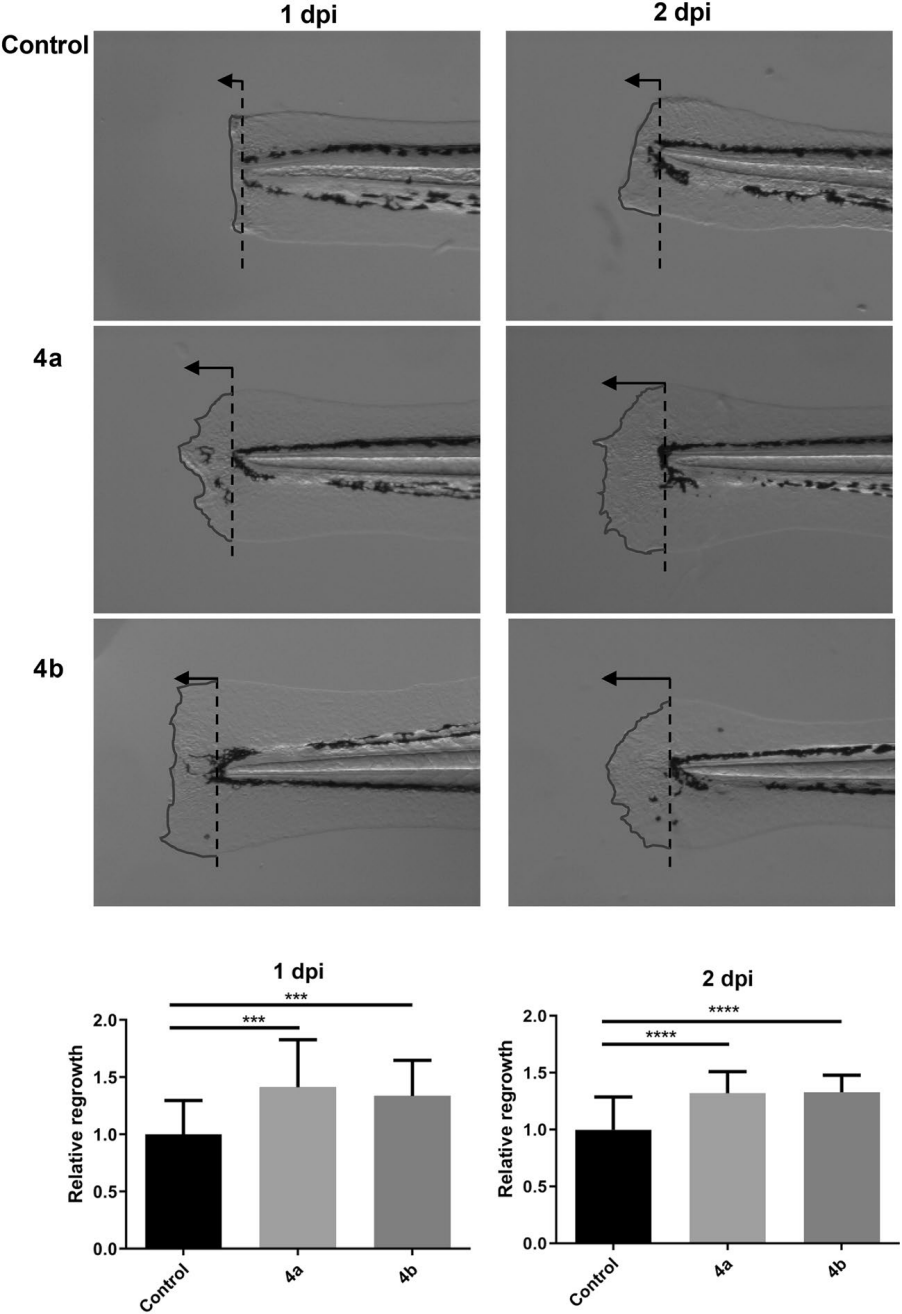
The in vivo impact of α-thio-β,γ-methylene-ATP on the tissue regeneration in Danio rerio model organism. (a) The images of tail zebrafish embryo were recorded by stereo microscope, dpi (day post incubation) represent the day after drug treatment. (b) The **4a** and **4b** promote the fin regrowth (n = 22, *** p < 0,001, **** p < 0,001). The regenerated parts of the tails have been marked with lines for better visibility.

Performed studies confirmed the influence of both diastereomers of α-thio-β,γ-methylene-ATP (**4a** and **4b**) on the tissue regeneration after tails cutting in *Danio rerio* studies (Fig. 7). In the untreated, control group, the regrowth of damaged fins in zebrafish embryos occurred but at a notably slow speed. In contrast, when animals were subjected to treatments with **4a** and **4b** for 1 and 2 days respectively, there was a significant enhancement in the growth of the tail fins. This compelling evidence indicates that **4a** and **4b** promote rapid tissue regeneration.

### In silico and in vitro analysis of possible interaction between tested ATP analogues and P2Y2 receptor

#### Molecular docking analysis of tested ATP derivatives indicated their interaction with P2Y2 receptor and revealed differences in interaction specificity of individual diastereomers of α-thio-ATP and α-thio-β,γ-methylene-ATP

For a better understanding of the differences in the results obtained for individual ATP analogues, a computational analysis of the interactions between the tested compounds and the P2Y2 receptor was performed. The P2Y2 receptor was modeled previously by Rafehi et al. in 2017^47^, however because the structure contains 2 flexible loops, it was necessary to verify whether there are any structural constraints in this region. For the homology modeling we used the SwissModel software (https://swissmodel.expasy.org)^40^ with the protein sequence obtained from the Uniprot database. The model was refined based on the P2Y1 receptor with high homology and the structure at the level of the intramembrane region was very similar (Fig. S18 left). As we compared the new model with the homology modeling published before by Rafehi et al.^48^ there was also high similarity with one difference in the variable loop of ATP binding region that was unstructured in previous models, but was somewhat structured and resolved here. The overall root mean-square deviation (RMSD) of atomic positions of P2Y1R (4XNW) and P2Y2R, which was homology modeled by Rafehi, was 0.219 Å. In the new model a partial β-sheet was formed between Thr174-Ala176 and Val181-Cys183 (Fig. S18). This might have an influence on the analysis of ATP and ATP analogues energy binding.

The molecular docking was done with the AutoDock Vina and AutoDock 4^49^. The grid box for docking was selected based on the ATP binding active center as it is in the P2Y1 receptor (Fig. S18). It was compared to the P2Y1 receptor that was crystalized (PDB 4XNW)^50^ with the 2-iodo-6-(methyl) adenine diphosphate derivative which may act as a good non-degradable ligand.

However, the analyzed previously P2Y2 receptor^47^ was used for further analysis. Here, the ATP was shown to interact with Arg177, Asp185, Arg265, Tyr268, Tyr269, Arg272, Lys289, and Arg292, and that surrounding was selected as the active center. The algorithm that was used in this study for molecular docking was Lamarckian Genetic Algorithm (LGA). LGA is a search-based method to estimate the optimal docking position between the ligands and the macromolecules, and then evaluates the results with an empirical binding free energy function. The genetic algorithm was based on the principles of biological evolutions through Genetics and Natural Selection. The molecular docking was done with the AutoDock Vina^51^ and AutoDock 4^49^.

**Figure 8:**
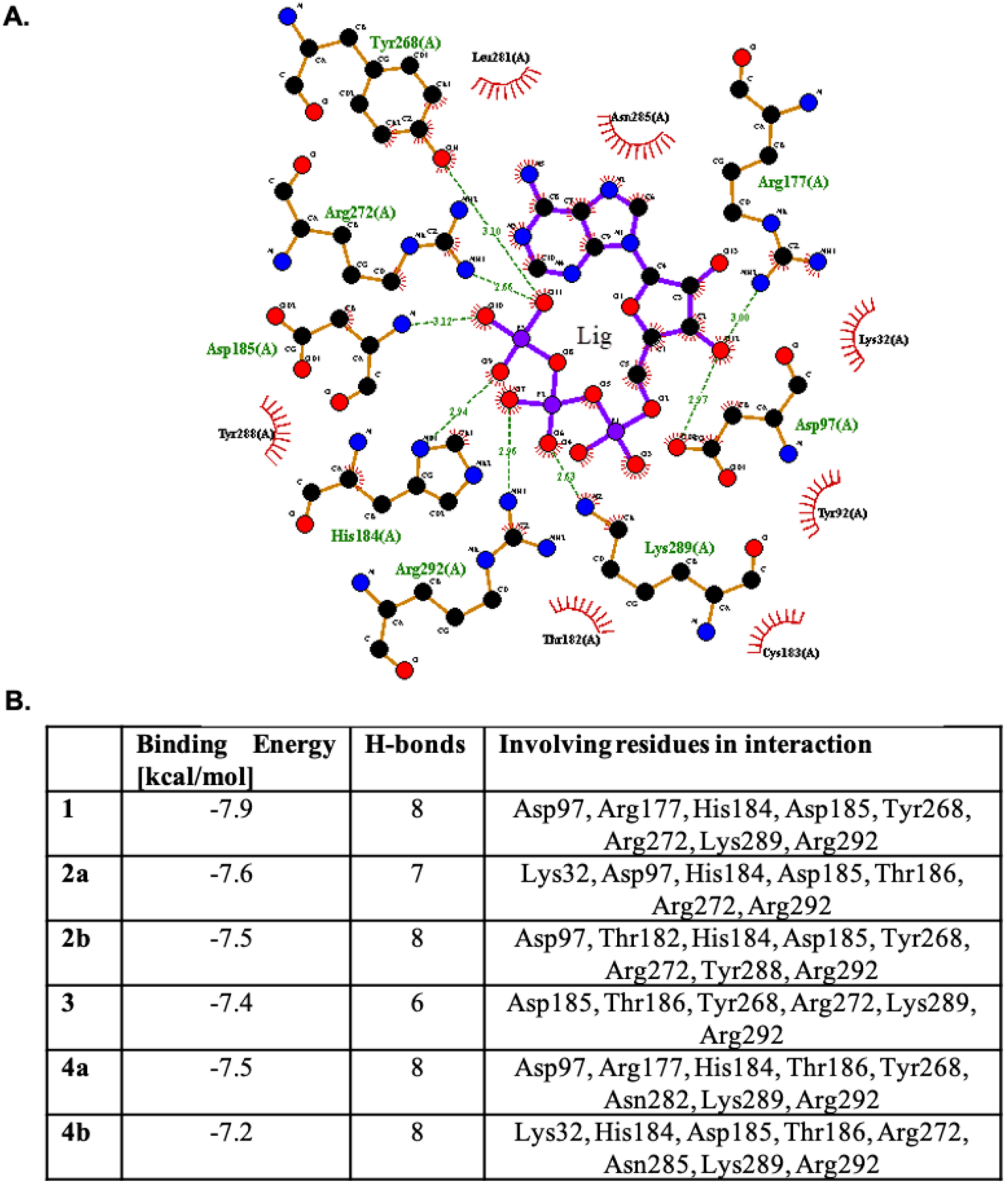
In silico studies on the interaction between ATP analogues and P2Y2 receptor. (**A**) LigPlot graph presents the 2D plot of P2Y2 receptor-ligand interactions with ATP (**1**) as a natural ligand. (**B**) The binding energy and number of hydrogen bonds calculated using molecular docking for ATP (**1**) and ATP analogues (**2a-4b**).

The binding energy of ATP and ATP analogues with P2Y2 receptor protein varied between -7.9 and -7.2 kcal/mol (Fig. 8). The ATP binding energy for the native ligand was the highest -7.9 kcal/mol and was similar to previous observations^47,52^. Also, the structure of ATP in the active center of the P2Y2 receptor was similar to the P2Y1 with RMSD = 0.107 Å and to the P2Y2 previously modeled by Rafehi et. al. 0.193 Å (Fig. 8). The binding energy for phosphorothioate derivatives (**2a** and **2b**) was slightly weaker than for unmodified ATP (**1**), however the *S*_P_ diastereomer (**2a**) has a stronger binding energy compared to its *R*_P_ counterpart (− 7.6 vs. − 7.5 kcal/mol, respectively).

Within the group of the β,γ-methylene-modified ATP derivatives (**3**, **4a** and **4b**), the most negative value of the binding energy was observed for the *S*_P_ diastereomer of α-thio-β,γ-methylene-ATP (**4a**), which had the stronger energy binding than its *R*_P_ counterpart (− 7.5 for *S*_P_ and − 7.2 for *R*_P_, respectively). Interestingly, both diastereomers of double-modified ATP analogues (α-thio-β,γ-methylene-ATP, **4a** and **4b**) occurred to create the same number of hydrogen bonds as the unmodified ATP (**1)** (8 hydrogen bonds in both cases), whereas in case of single-modified β,γ-methylene-ATP (**3**), the observed number of H-bonds was only 6 (Fig. 8). The lower value of H-bonds for compound **3** compared to other tested ATP analogues may suggest the weaker interaction with P2Y2 receptor and provide an explanation for the lack of induction of purinergic signaling in P2Y2 expressed HaCaT cells.

The interaction of ligands with the receptor is presented in the LigPlot graphs in details here in Figs. 8 and S19. LigPlot is a program for automatic generation of 2D ligand–protein interaction diagrams with the set of interacting residues. Using this analysis, it was shown, that when comparing ATP (ligand **1**) and methylene derivative (ligand **3**), there were only 5 similar residues involved in the interaction (Asp185, Tyr268, Arg272, Lys289, Arg292), whereas when comparing ATP (**1**) and the most active α-thio-β,γ-methylene-ATP analogue (**4a**) we have observed interaction of ligand with 6 amino acids residues (Asp97, Arg177, His184, Tyr268, Lys289, Arg292), which suggest that ligand **4a** is more similar to the ATP as the potential agonist of P2Y2 receptor with a comparable net of interactions (Figs. 8 and S19). Interestingly, in case of compound **3**, which occurred to be inactive in induction of purinergic signaling (Fig. 4)**,** the interaction with His184, which is known to be one of the amino acids involved in the interaction with phosphate group of the ligand^53^, was not detected.

#### The P2Y2 antagonist-based studies confirmed interaction of α-thio-modified ATP analogues with the P2Y2 receptor

**Figure 9:**
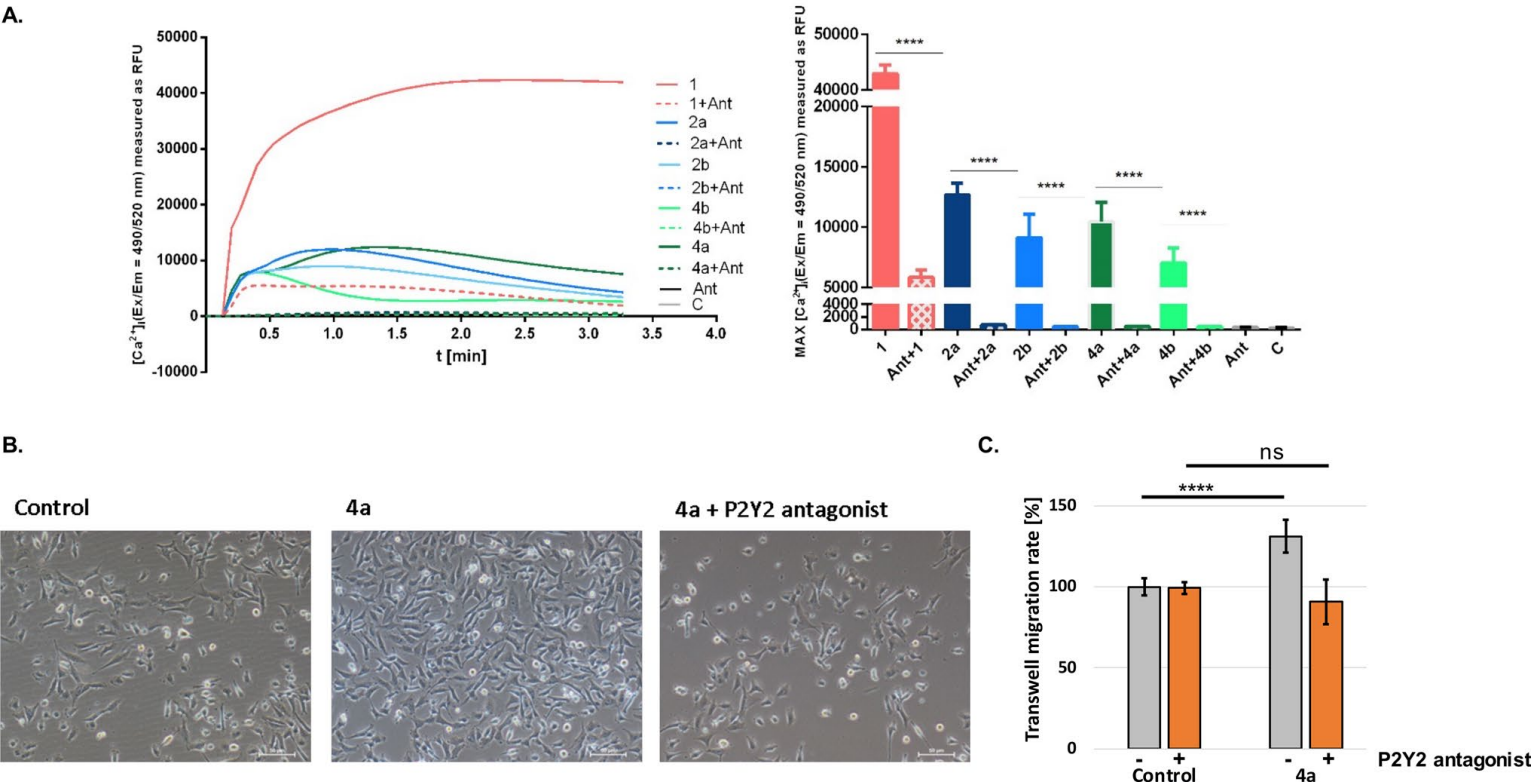
In vitro verification of ATP analogues activity in the presence of P2Y2 receptor antagonist. (**A**) The impact of tested ATP analogues on Ca^2+^ mobilization in HaCaT cells in the presence and absence of P2Y2 antagonist (**B**) The influence of **4a** derivative on transwell migration of MDA-MB-231 cells in the presence and absence of P2Y2 antagonist. Data represent the means ± SEM from at least 3 independent experiments. ****p < 0.0001 vs control. C—untreated control cells, RFU—relative fluorescence units. Scale bars 50 μm.

Based on the in silico analysis it could be concluded, that the interaction between tested α-thio-modified ATP analogues and P2Y2 receptor is possible. In order to verify experimentally whether synthesized ATP analogues may interact with the P2Y2 receptor, the selective P2Y2 antagonist—AR-C118925XX was used. It was shown, that after using a P2Y2 receptor antagonist, the intercellular calcium mobilization induced by the α-thio-modified ATP analogues was almost completely eliminated (Fig. 9A). Such results obtained in the presence of P2Y2 selective antagonist, clearly confirmed, that all of the tested α-thio-modified ATP derivatives (**2a**, **2b, 4a** and **4b**) might act as agonists of the P2Y2 receptor.

Furthermore, functional analysis revealed that the increase in the transwell migration rate of MDA-MB-231 cells induced by the highly active α-thio-β,γ-methylene-ATP (**4a**) was abolished in the presence of the P2Y2 receptor antagonist (Fig. 9B and C). Obtained data confirmed the influence of the interaction with the P2Y2 receptor on the the promigratory activity and the ability to induce purinergic signaling of *S*_P_-α-thio-β,γ-methylene-ATP.

## Discussion

Nowadays, various strategies are employed to accelerate wound healing and tissue regeneration^5^. Dressings coated with various antibacterial or wound healing substances are often used for this purpose^54,55^. One of them is application of nucleotides themselves^8,35,56^ or materials for their further synthesis^54^. Induction of purinergic signaling based on nucleotides from the microenvironment of the healing wound is one of the approaches to improve chronic wound healing. It has been shown, that extracellular nucleotides might be involved in the activation of cellular pathways responsible for proper wound healing via interaction with surface receptors^8,56^, what makes them a good target for rational designing of new analogues for enhancement of this communication.

It was demonstrated, that the most highly expressed purinergic receptor in human keratinocytes is P2Y2^10,21,57^ and activation of this receptor occurs during wound healing^8,56^. Furthermore, the release of endogenous ATP into the extracellular space, and its further interaction with P2Y2 has been proposed as an autocrine signaling mechanism to facilitate wound closure^8^. Thus, the P2Y2 receptor seems to be a promising target for the development of innovative approaches in wound healing therapy. Based on this data, we proposed double-modified α-thio-β,γ-methylene-ATP analogue as potential ligands for the P2Y2 receptor, which through its stimulation could enhance tissue regeneration processes. Performed studies were focused on the verification whether the double-modified α-thio-β,γ-methylene-ATP analogues would be better than their single-modified counterparts (α-thio-ATP and β,γ-methylene-ATP), and would have the ability to interact with the P2Y2 receptor and thus whether these compounds are able to induce the purinergic signaling, which is known to activate processes related to skin regeneration. To date, several different methods have been developed for the synthesis of nucleoside triphosphate, both natural as well as their modified analogues^58–60^. Recently, the use of the P(V) platform for the stereocontrolled synthesis of symmetrical as well as unsymmetrical dinucleoside thiodiphosphates and thiotriphosphates was presented by Baran^61^. However, the oxathiaphospholane approach also provides a convenient method for the synthesis of modified NTP analogues. Here, its usefulness for the synthesis of non-hydrolyzable ATP analogues that are doubly modified within the phosphate residue has been presented. All tested α-thio-modified ATP analogues i.e. double modified α-thio-β,γ-methylene-ATP, as well as single modified α-thio-ATP was synthesized in form of P-diastereomerically pure isomers using OTP method, in which the protected adenosine 5′-O-(2-thio-1,3,2-oxathiaphospholane) reacted with pyrophosphate or methylene diphosphonate, in the presence of DBU with moderate yields, comparable to other synthetic methods.

Both, theoretical and experimental studies conducted as part of this research confirmed the interaction of all analyzed α-thio-modified ATP analogues with P2Y2 purinergic receptor indicating importance of this interaction on their biological activity. It is known, that extracellular ATP can affect the intracellular free Ca^2+^ concentration through the purinergic signaling, which is linked to several processes, including wound healing^10^. Certainly, ATP-sensitive purinergic receptors form a system of cell-surface proteins that become active upon binding to ATP. ATP serves as the natural activator for all P2X receptors, along with P2Y2 and P2Y11 receptors. When stimulated simultaneously by ATP, each subtype of P2 receptors contributes to a rise of a complex calcium response. This complexity is further heightened by the fact that each ATP-binding receptor has distinct affinities for ligands and produces diverse effects on plasma membrane calcium pumps. Concerning α-thio modification, based on the results obtained for thio-modified nucleoside monophosphates, such as thymidine-5′-O-monophosphorothioate (TMPS), which has been shown as an active compound in promotion of keratinocytes migration and stimulation of in vivo angiogenesis^57^, we have assumed that α-thio-modified ATP analogues are worth to analyzed for their potential pro-regeneration activity. Obtained result not only confirmed the agonist activity for P2Y2 receptor and ability to increase of [Ca^2+^]_i_ mobilization of α-thio-modified ATP derivatives, but also revealed some differences between individual diastereomers. In both pairs, α-thio-ATP as well as α-thio-β,γ-methylene-ATP more active appeared to be *S*_P_ diastereomer. This observation was in accordance with previous studies, where better recognition, reactivity and biological activity of *S*_P_ diastereomers of α-thio-modified ATP analogues was observed for various ATP-dependent targets^31,39^.

Interestingly, single modified β,γ-methylene-ATP occurred to be not active in the intercellular calcium mobilization in human keratinocytes. Since in the context of wound healing, the most important function of ATP seems to be its involvement in the purinergic signaling in the wound area, we focused on other analogues for further study. Although, β,γ-methylene-ATP-induced calcium release is negligible, its α-thio modified counterparts (**4a** and **4b**) were able to elicit calcium release in a P2Y2-dependent manner. The in silico analysis of P2Y2 interaction with β,γ-methylene-ATP and α-thio,β,γ-methylene-ATP revealed differences in energy binding and involvement of amino acid residues available in the receptor binding site that could interact with the ligand. The smallest similarity to the natural ligand and the lowest number of possible hydrogen bonds were observed for β,γ-methylene-ATP, which may explain its lack of activation of purinergic signaling in P2Y2-rich human keratinocytes. Since P2Y2 purinergic receptor has been indicated as the highly expressed in human keratinocytes and involved in the skin regeneration processes^8,57^, the interaction of nucleotide analogues with this target seems to be a crucial for their possible impact on wound healing, however the precise mechanism remains to be elucidated.

Stimulation of keratinocytes migration is indicated as a key factor, which may facilitate wound healing^62^. This effect has been shown to be related to the induction of epithelial–mesenchymal transition (EMT), partial EMT but also collective cell migration^6,63^. Literature data show that interaction of ATP with P2Y2 result in intracellular Ca^2+^ mobilization, and then enhancement of keratinocytes migration, without affecting the cells proliferation^8,64^. The similar effects have been observed in current work for the *S*_P_-α-thio-β,γ-methylene-ATP (**4a**) derivative, which is proposed as a pro-regeneration agent. In performed research, we also noted increase of keratinocytes migration without affecting cell viability, increase of [Ca^2+^]_i_ mobilization and induction of EMT after treatment with this compound. Further analysis using P2Y2 selective antagonist confirmed the dependence of [Ca^2+^]_i_ mobilization on the interaction with the P2Y2 receptor in human keratinocytes. Based on these data, it can be concluded, that the mechanism of action of **4a** derivative might be similar to ATP, the natural P2Y2 ligand. Additionally, after using this derivative simultaneously with selective P2Y2 antagonist, the calcium mobilization was almost completely abolished. Whereas in case of ATP under the same conditions, partial activity could still be observed, probably as a result of ATP interaction with other ATP-dependent targets.

Besides the impact on the keratinocytes, literature data show, that extracellular ATP may enhance migration and induce epithelial–mesenchymal transition (EMT) related changes via the interaction with P2Y2 receptor, also in other types of cells with a high expression of this receptor, like breast cancer cells^45,46,65^. Therefore, the MDA-MB-231 as one of the highly P2Y2- expressed type of cells has been chosen as a convenient model for assessment of impact of α-thio-β,γ-methylene-ATP derivatives on the induction of EMT-related processes. Also, in this analysis, a proposed pro-regeneration agent occurred to be active. Both diastereomers of α-thio-β,γ-methylene-ATP have been shown to increase expression of mesenchymal markers, enhance transwell migration of treated cells, as well as increase the ability of crossing through collagen barrier. Numerous data show, that the epithelial–mesenchymal transition (EMT) and extracellular matrix (ECM) remodeling involving endopeptidases such as matrix metalloproteinases (MMPs) are the crucial processes for wound healing and tissue regeneration^44,66^. The main activity of matrix metalloproteinases is the degradation of the collagen barrier, which results in the enhancement of cell migration and invasiveness. The activity of these collagenases occurred to affect wound healing^7^. Thus, the involvement of α-thio-β,γ-methylene-ATP derivatives in the increased expression of EMT markers, combined with enhancement of cell migration and their ability to cross the collagen barrier make this ATP analogues great candidates for consideration as agents supporting regenerative processes. The final confirmation of this thesis is the result of the in vivo analysis, where the enhancement of cutting *Danio rerio* tails in the presence of α-thio-β,γ-methylene-ATP has been demonstrated.

Although, in Zebrafish-based in vivo studies beside *S*_P_-α-thio-β,γ-methylene-ATP derivative (**4a**), also its *R*_P_ counterpart (**4b**) exhibited pro-regeneration activity, the difference in pro-regeneration potential between *R*_P_ and *S*_P_ diastereomers in the human skin cells was significant. The differences in the activity of individual diastereomers might be caused by their various specificity of interaction with the P2Y2 target in the tested human cells. For both pairs of diastereomers, the energy binding for *S*_P_ diastereomers was lower compared to the value for *R*_P_. This result might explain the greater activity of the *S*_P_ diastereomers in the acceleration of cell migration for *S*_P_-α-thio-β,γ-methylene-ATP (**4a**), as well as for *S*_P_-α-thio-ATP (**2a**) in the HaCaT cells which are known for the high expression of P2Y2 receptor. Considering all the data, double-modified α-thio-β,γ-methylene-ATP diastereomers were tested in vivo and in model MDA-MB-231 cells. All obtained data confirmed pro-regeneration potential of the *S*_P_-α-thio-β,γ-methylene-ATP (**4a**), which is proposed as the promising agent for the skin regeneration applications and as a drug candidate for the pro-regeneration applications, which may enhance wound healing in P2Y2 dependent manner via induction of epithelial–mesenchymal transduction and increasing migration rate of human keratinocytes.

## Materials and methods

### Synthesis of ATP derivatives

#### General remarks

Adenosine was purchased from Pharma Waldhof (Germany). The acetic anhydride,1,4-diazabicyclo[5.4.0]-undec-7-ene (DBU), phosphorus trichloride, as well as 1,2-ethanediol and tris(tetrabutylammonium) hydrogen pyrophosphate and methylenediphosphonic acid were purchased from Sigma-Aldrich/Merck (USA). Chloroform, triethylamine and methanol were provided by POCH (Poland). Elemental sulphur was dried under high vacuum for 12 h. Acetonitrile (HPLC grade, JT Baker), which was used as a solvent for ring opening reaction, was stored over 3 Å molecular sieves until the residual moisture content dropped below 10 ppm (by Karl-Fischer coulometry). The obtained adenosine 5′-*O*-(1-thiotriphosphate) analogues were separated into P-epimers using a binary Varian HPLC system, consisting of two PrepStar 218 pumps and a ProStar 325 UV/VIS detector set at 260 nm. A reverse phase HPLC column (PRP-1, C18, 7 mm, 3057 mm, Hamilton, Reno, NV) was eluted with a gradient of CH_3_CN (1% min^−1^) in 0.1 mol/L TEAB (pH 7.3) at a 2.5 mL min^−1^ flow rate.

Analytical RP-HPLC were performed using Kinetex^®^ 5 mm column 100 A (4.6 250 mm, Phenomenex) at 1 mL min^−1^ flow rate; buffer A, 0.05 mol/L triethylammonium bicarbonate (TEAB) buffer pH 7.3; buffer B, 40% CH_3_CN in 0.05 mol/L TEAB; a gradient 0 to 40% B over 30 min. The open column chromatographic purification was performed using Silica gel 60, 200–300 mesh. TLC silica gel 60 plates with a UV F254 indicator, were used for routine analyses^39^. Silica gel chromatography media were purchased from Merck.

#### Analytical methods

The ^1^H NMR and ^31^P NMR spectra were recorded using Bruker AV- 200 (200 MHz for ^1^H, 81 MHz for ^31^P) or DRX-500 (500.13 MHz for ^1^H, 202.46 MHz for ^31^P) instruments, with TMS or 85% H_3_PO_4_ as external standards. High-resolution mass spectra (HRMS) were recorded using a Synapt G2 Si mass spectrometer (Waters) equipped with an ESI source and a quadrupole-time-of-flight mass analyzer. The measurements were performed in negative or positive ion modes, with the capillary and sampling cone voltage set to 2.7 kV and 20 V, respectively. The source temperature was 110 °C. To ensure satisfactory accuracy, data were collected in a centroid mode and the readings were corrected during acquisition using leucine enkephalin as an external reference (Lock-SprayTM), which generated the reference ions at m/z 554.2615 Da ([M−H]^−^) in the negative ESI mode and at m/z 556.2771 Da ([M+H]^+^) in a positive ESI mode. The data sets were processed using the MassLynx 4.1 software (Waters).

#### Synthetic procedures

The sodium salts of pyrophosphate analogues were converted to tri-n-butylammonium salt by elution of an aqueous solution of the appropriate sodium salt through the DOWEX hydrogen form cation exchange resin according to the previously described procedure^33,39,41^. The solution was then lyophilized. The 2′,3′-*O*-diacetyladenosine 5′-*O*-(2-thio-1,3,2-oxathiaphospholane) (0.25 mmol) and the appropriate phosphate tri-n-butylammonium salt (0.5 mmol) were dried together overnight over P_2_O_5_ under vacuum. Next, the flask was filled with dry argon and the solution of DBU (0.5 mmol) in anhydrous CH_3_CN (3 mL) was added dropwise through the septum using a Hamilton syringe. After 3 h at room temperature the mixture was concentrated under reduced pressure. The protecting acetyl groups were removed from 2′- and 3′-hydroxyl groups by treatment with 25% aqueous ammonia. The obtained ammonia solution was then evaporated to dryness. Then, the product was purified by ion exchange chromatography (DEAE-Sephadex A-25 column). Chromatography was performed with a linear gradient of 1 L each 0.01 and 0.6 mol/L triethylammonium bicarbonate buffer (TEAB). Fractions containing product were collected and evaporated to dryness on a rotary evaporator. The remaining residue was co-evaporated twice with methanol (50 mL) to remove traces of the buffer. Finally, the product was lyophilized in a high vacuum. The yields and physicochemical characteristics of the compounds are summarized in the supplementary materials.

### Cell culture

The immortal human keratinocyte HaCaT cell line (DSMZ; ACC-771) was purchased from Leibniz Institute DSMZ-German Collection of Microorganisms and Cell Cultures (Braunschweig, Germany). The MDA-MB-231 (human breast adenocarcinoma) were purchased from Cell Biolabs (San Diego, California, USA, cat no AKR-201). HaCaT cells were cultured in Dulbecco’s modified Eagle’s medium (DMEM) (Biological Industries) with 10% fetal bovine serum (FBS) (Sigma-Aldrich, St. Louis, MO) and antibiotics (100 U/mL penicillin and 100 μg/mL streptomycin (BioWest)). The medium for MDA-MB-231 cells were additionally supplemented with l-glutamine and non-essential amino acids. All cells were cultured under standard conditions (37 °C, 5% CO_2_) and were passaged twice a week. Prior to the usage, cultures were tested for the presence of mycoplasma infection to ensure mycoplasma free conditions. Tests were performed using EZ-PCR Mycoplasma Test Kit with internal control (Biological Industries, Israel). For each experiment, cells were counted using ScepterTM 2.0 Cell Counter (Merck, Darmstadt, Germany).

### Cytotoxicity test

Prior to the experiments, cells were seeded into 96-well plates at a concentration of 10,000 cells per well and allowed under standard conditions (37 °C, 5% CO_2_) to adhere to the plate surface. The next day, the culture medium was removed and a fresh medium containing tested compounds was added. After a 24 h incubation, the (3-(4,5-dimethyl-2-thiazolyl)-2,5-diphenyltetrazolium bromide (MTT reagent) was added to the culture medium and plates were left for additional 2 h at 37 °C. Then, the culture medium was removed, and 100 µL of isopropanol was added to each well. Plates were shaken for 1 h at room temperature. After that, the absorbance was measured at 570 nm and 630 nm (reference value) using a Synergy HT plate reader (Bio-Tek, Winooski, Vermont, USA) and KC4 3.2 Rev 2 software (Bio-Tek, Winooski, Vermont, USA). Tested compounds were used at concentrations of 100 µM. For the verification of the test correctness, 1% SDS solution was used. The cytotoxicity level of each compound was calculated relative to the negative control (untreated cells) set as 100%. Each viability point represents the mean value from at least three independent experiments. Individual variants were repeated at least three times in each experiment.

### Calcium mobilization assay

HaCaT cells were seeded into 96-well plates at a concentration of 30,000 cells per well and cultured for 24 h (37 °C, 5% CO_2_). Subsequently, the culture medium was changed to a Ca5 buffer. The intracellular calcium concentration [Ca^2+^]i was measured with the Screen QuestTM Fluo-8 No Wash Calcium Assay Kit according to the producer’s protocol. Calcium flux was measured after stimulation with ATP analogues at 100 µM concentration and/or P2Y2 antagonist AR-C 118925XX (Tocris Bioscience) applied at a concentration of 10 µM by the change in the fluorescence (excitation/emission = 490/520 nm) measured on a Synergy 2 microplate reader (BioTek, Winooski, VT, USA). Fluorescence reads were corrected for background fluorescence. The MAX fluorescence was read from the real-time kinetics of [Ca^2+^]i quantitative data and calculated in GraphPad, which selects the first maximum peak after the addition of the test compound for each measurement and then averages the result. Statistical analysis of the data was performed using 1way ANOVA and 2way ANOVA tests.

### Wound healing assay

For the migration studies, HaCaT cells were seeded into 24-well plates at a concentration of 500,000 cells per well and allowed to adhere in standard conditions (37 °C, 5% CO_2_) for 24 h. Then, the scratches were prepared in each well. Cells were washed thrice with PBS buffer, and a new medium containing tested compounds at the final concentration of 100 μM was added. Cells were then cultured with tested compounds in a cell culture incubator under standard conditions (37 °C, 5% CO_2_). The microscopic observations and measurements of the sizes of wounds were performed immediately after adding tested compounds (time 0), and subsequently after 6 h and 24 h of incubation using a Nis Eclipse Ti microscope (Nikon, Tokyo, Japan). Data were recorded and analyzed using Nis-Elements BR 4.30.00 software (Nikon).

Analysis for individual samples after incubation with the tested compounds was performed based on the initial scratch size in each well. The data obtained in this way were then compared with the control sample (untreated cells). The mean size of cell free gap in the wells without tested compounds was used as a control and set as 100%. The influence on the migration rate was estimated based on the size of the wound area after compound treatment related to the control sample.

### Transwell migration of MDA-MB-231 cells

Transwell migration studies were performed using 24-well plates (Corning) and transwell migration inserts with 8 μm diameters pore sizes (Corning). The complete cell culture medium containing Dulbecco’s modified Eagle’s medium (DMEM) (Biological Industries) supplemented with 10% FBS, l-glutamine, non-essential amino acids, 100 U/mL penicillin and 100 μg/mL streptomycin (BioWest) were added into each well. Then MDA-MB-231 cells were seeded into the transwell chambers in the serum-free medium containing tested compounds at the concentration of 100 μM. Plates were then incubated in a humidified tissue culture incubator at 37 °C, 5% CO_2_ for 72 h. After this time, inserts were removed and each well of the plate was washed by PBS. The number of cells that passed through the pores in the membrane to the lower chamber was analyzed by microscopic observation and crystal violet staining.

### Passing through the collagen barrier

For the assessment of cells’ ability to penetrate through the collagen barrier in the presence of tested compounds, the QCM Collagen Cell Invasion Assay (Merck, Saint Louis, MO, USA) was used. Tests were performed according to the manufacturer's protocol using MDA-MB-231 cells as a model cell line for invasion studies. The 10% FBS was used as a chemoattractant. The ability to migrate through the collagen layer was assessed based on the fluorescent labelling of passed cells using CyQuant GR dye (Merck, Saint Louis, MO, USA). The measurements were performed using a Synergy HT plate reader (Bio-Tek, Winooski, Vermont, USA) with KC4 3.2 Rev 2 software. Results obtained for the cells cultured in the presence of 100 μM ATP and ATP analogues: 4a, 4b were calculated in the comparison to the control sample (untreated cells).

### Immunostaining of cells

After treatment with tested compounds, MDA-MB-231 or HaCaT cells were fixed using 3,8% paraformaldehyde and washed thrice with PBS. Permeabilization was performed by incubation the samples with 0,01% Triton X-100 in PBS. Incubation was performed for 10 min at room temperature with gentle agitation. Then, cells were washed three times with PBS and blocked using 10% serum for 1 h at room temperature. For detection of vimentin cells were incubated with primary rabbit anti-vimentin antibodies (D21H3, Cell signaling) at 4 °C overnight. After thrice washing, cells were treated with secondary anti-rabbit Alexa-Fluor-conjugated antibodies (Abcam Cambridge, UK).

### DAPI staining

Immunofluorescent labeled cells were stained for 10 min with 4′,6-diamidino-2-phenylindole (DAPI) (Sigma Aldrich, St. Louis, MO) solution in PBS at a final concentration of 5 μg/ml. Incubation was performed at room temperature in the dark. After staining cells were washed three times with PBS. Microscopic observation was performed using a Nikon Eclipse microscope with appropriate optical filters. The images were performed and analyzed using NisElement software (Nikon).

### Assessment of epithelial–mesenchymal transition in model Zebrafish system

#### Zebrafish maintenance

The Fli:GFP/Casper zebrafish line (*Danio rerio*) lacks pigmentation and its vascular endothelial is labelled with GFP. This allows for non-invasive in vivo imaging in larvae which are transparent during the entire research time (up to 8 days post fertilisation). In order to breed larvae, 30 adult fish were divided over 3 tanks with a sloping mesh at the bottom. Overnight, the fish mated, and eggs had dropped through the sieve. Adult fish were placed back into their tank. Subsequently, eggs were cleaned with egg water (Instant Ocean^®^ Sea Salt) and placed in Petri dishes with egg water inside an incubator at 28 °C. The next day, at 1 day post fertilisation (dpf), dead eggs were removed, and egg water was refreshed. This was repeated at 2 dpf, after which the larvae were moved to a 33 °C incubator. Since tumour cells grow optimally at 37 °C and zebrafish at 28 °C a compromise was made, and the larvae were kept at 33 °C.

#### Xenografting

First, cells were digested for 5 min with 1 ml of 10% Trypsin solution at 37 °C 5% CO_2_. Digestion was stopped by addition of 4 ml fresh DMEM 10% FCS. Then, 10 μl of cells was mixed with 10 μl Trypan Blue (Thermo Fischer Scientific) and 10 μl of this mix was added to a dual-chamber cell counting slide (BIO-RAD). The number of live cells was counted using BIO-RAD Automated Cell Counter TC20. Cells in solution were centrifuged at 250×*g* for 5 min. After that, supernatant was removed and 1 ml PBS/EDTA was added. The tube was vortexed to bring the cells back into suspension and another centrifugation step was performed. Then, the supernatant was removed again and the cells were suspended in a 2% polyvinylpyrrolidone-40 (PVP, CalBioChem) solution. Needles were created for injection by heating and pulling borosilicate glass tubes (outer diameter 1.0 mm, inner diameter 0.78 mm, Science Products). Next, under a stereo microscope, the tips of pulled glass tubes were cut off with tweezers, creating needles. The larvae were brought under anesthesia by adding 2.5 ml 20% Tricaine to the egg water. To inject larvae, needles were loaded with 2*10^8^ cells/ml, and using a picopump 1 nanoliter containing 300–500 tumour cells was injected into the Duct of Cuvier in each larva. Injected larvae were placed in a petri dish with fresh egg water and placed in incubator at 33 °C. The 50 larvae per each group were injected. This experiment was repeated to compensate for genetic variation since the crossed parent fish are not clonal.

#### Maximum Tolerated Dose (MTD) for wild-type zebrafish

To determine the MTD of **4a** and **4b** solutions in wild-type zebrafish, solutions of 1 μM, 5 μM, 10 μM, 20 μM, 50 μM and 100 μM were prepared before the experiment. At 2 dpf, the drugs were added to the fish water and renewed at the same concentrations. To determine the MTD of zebrafish, images of treated and wild-type embryos at 6 dpf were taken using a DFC420C camera coupled to a Leica MZ16FA fluorescence microscope.

#### Effects of tested compounds in a zebrafish ectopic tumor model

For in vivo tracking of cells migration, the fluorescent PC3Pro4 and MDA-MB-231 cells were used. The PC3Pro4 cells were kindly provided by Dr. Gabriel van der Pluijm (Department of Urology, LUMC), whereas MDA-MB-231 cell line (RRID:CVCL_0062) were originally obtained from American Type Culture Collection (ATCC). Cells were injected at 2 dpf into the Güvenberg catheter (ectopic model) and the drug was delivered via WA. For WA delivery, 20 μM **4a** and 20 μM **4b** solutions were added at 2 dpf to zebrafish injected with tumor cells and maintained until 6 dpf. Fish water was refreshed with drug at 3, 4, 5 and 6 dpf. After treatment, images of embryos were acquired using a Leica M165 FC stereo fluorescence microscope. Tumor metastasis was quantified by calculating the ratio of fluorescence intensity of extravasated tumors at the zebrafish CHT (Leiden). Each experiment was performed at least 3 times with group size > 30 embryos.

### qPCR analysis of EMT markers

#### RNA extraction and qPCR analysis of EMT markers from in vitro cultured cells

Cells were collected and counted, and then 500 μl of Trizon (15596026, Invitrogen) was added to the 10^6^ to 10^7^ number of cells to lyse them. Cells were suspended in Trizon and incubated at room temperature for 5 min. After that, 0.1 ml chloroform was added to the lysis and probes were incubated for additional 3 min at room temperature. Then samples were centrifuged 12,000*g* for 15 min at 4 degrees and the colorless liquid layer containing RNA was collected from the layered liquid. Next, 0.25 ml of isopropanol was added to the RNA-containing liquid and samples were mixed. After incubation at room temperature for 10 min, the centrifugation was performed again at 12,000*g* for 10 min at 4 °C. After centrifugation, the supernatant was removed and 1 ml of 75% ethanol was added to gently resuspend the RNA. After another centrifugation at 7500*g* for 5 min at 4 °C, the ethanol was removed and the purified RNA was air-dried at room temperature. Isolated RNA was resuspended by addition of 30–50 μl of RNAase-free water and incubation at 55–60 °C for 10 min. The concentration of the extracted RNA was calculated, and DNase was added to remove the DNA in the sample, and then the RNA was reverse-transcribed into cDNA using the iScript Reverse Transcription Super Mix Kit (1708841, Bio-Rad). Intracellular expression of different genes was detected by real-time PCR (Universal SYBR Green Supermix, 1725270, Bio-Rad).

#### RNA extraction and qPCR analysis of EMT markers from in vivo Zebrafish experiments

Zebrafish with tumor cells samples were collected by cutting of the entire tail of zebrafish (70 fish per group) at 6 dpi using micro dissecting scissors (WPI, FL, USA). Immediately after cutting, samples were put into TRIzol (Sigma-Aldrich, Zwijndrecht, Netherlands). The whole process was completed within 30 min. Whole RNA was isolated using the RNeasy Mini Kit (Qiagen) according to the manufacturer's protocol. The iScript™ cDNA Synthesis Kit (Bio-Rad, Utrecht, The Netherlands) was used for cDNA synthesis and iQ™ SYBR^®^Green Supermix (Bio-Rad, Utrecht, The Netherlands) was used as described in the manufacturer's protocol for qPCR. For each gene analysis, human-specific primers were designed to measure gene expression variation in human cancer cells. Species specificity was tested prior to the experiments. The expression of *GAPDH* gene was treated as a control sample and was used as a reference for calculation the relative expression of EMT genes in the samples.

#### Zebrafish regeneration studies

The wound in 2 dpf embryos was induced by cut off of the fin from the tail of the zebrafish^67,68^. The zebrafish with the fin cut off were incubated with egg water (control group) or 20 μM of **4a** or **4b** compounds added to egg water immediately after wounding. The regrowth of the zebrafish fin was recorded by microscopy after 24 h and 48 h of incubation, respectively. The length of the tail fin growth was calculated by Image J for different groups of zebrafish.

#### Computational analysis of interaction between ATP derivatives and P2Y2 receptor

The structure for the P2Y2 receptor was modeled using SwissModel software. For that the FASTA sequence of the receptor was obtained from uniport database (P41231). The 3D model was prepared in the SWISSMODEL (https://swissmodel.expasy.org)^48^. The ATP model was acquired from PDB database. The ligands derivatives were prepared based on the ATP compound by using ChemDraw software (https://perkinelmerinformatics.com). For the next step the unmodified ATP and ATP analogues were selected as ligands for molecular docking with the P2Y2 receptor protein. The molecular docking and binding energy evaluation for the obtained structures, was done using AutoDock Vina and AutoDock 4.2.6^49^. Molecular docking calculation using AutoDock was performed in several steps: (1) preparation of coordinate files using AutoDockTools, (2) pre-calculation of atomic affinities using AutoGrid, (3) docking of ligands using AutoDock, and (4) analysis of results using AutoDockTools. The structure for the P2Y2 receptor protein, obtained by homology modeling, and ATP analogues was prepared as follows.

Step 1: Coordinate file preparation. The structure for P2Y2 receptor protein was prepared by adding polar hydrogens, charges and saved in the PDBQT file format. The unmodified ATP and ATP analogues, were prepared in similar fashion by adding polar hydrogens, charges and saved in separate PQBQT files. PDBQT was used for coordinate files, which includes atomic partial charges and atom types. PDBQT files also include information on the torsional degrees of freedom.
Step 2: AutoGrid calculation. In the AutoGrid procedure, the protein was embedded in a three-dimensional grid and a probe atom is placed at each grid point. The grid box was selected based on the ATP binding active center in the size of 40 × 40 × 40A that was saved in the GPF file. AutoGrid affinity grids were calculated for each type of atom in the ligand, typically carbon, oxygen, nitrogen and hydrogen, as well as grids of electrostatic and desolvation potentials.
Step 3: Docking using AutoDock. Docking was carried out using Lamarckian genetic algorithm (LGA). AutoDock was run several times to give several docked conformations, and analysis of the predicted energy and the consistency of results was combined to identify the best solution.
Step 4: Analysis using AutoDockTools. For analyzing the results of docking simulations, interactions between P2Y2 receptor protein, ATP and ATP analogous, and the affinity potentials created by AutoGrid were visualized.

### Ethics approval

All animal experiments were approved by the Animal Experiments Committee (Dier Experimenten Commissie, D.E.C.) under license AVD1060020172410 (valide from 16 januari 2018 to 1 januari 2023) and licence AVD10600202216495 (from 11 januari 2023 to 10 januari 2028). All animals were maintained in accordance with local guidelines using standard protocols: http://www.ZFIN.org (accessed on 12 December 2019). All experiments were performed in accordance with relevant guidelines and regulations. The authors complied with the ARRIVE guidelines.

## Conclusion

Extracellular nucleotides are indicated as important signaling molecules that may facilitate skin regeneration processes via interaction with the P2Y2 receptor. Herein, double-modified analogue of ATP—α-thio-β,γ-methylene-ATP is proposed as a potential therapeutic agent that can facilitate regeneration processes. Since mentioned ATP derivative occurs in the form of two molecules that differ only in their spatial structure – diastereomers *S*_P_ and *R*_P_ – in order to test whether the individual diastereomers could differ in their activity, the procedure of synthesis and separation of the reaction mixture was prepared in such a way as to obtain pure diastereomers. Due to this approach, it was possible to perform a detailed analysis of the pro-regeneration potential of two (*S*_P_ and *R*_P_) diastereomers of tested compound. The in vivo studies have shown that α-thio-β,γ-methylene-ATP derivatives significantly enhance regeneration of cut Zebrafish tails. Detailed in vitro studies on human keratinocytes and MDA-MB-231 model cells confirmed their involvement in P2Y2-dependent calcium mobilization and induction of EMT-related processes. Furthermore, by using separated diastereomers of this ATP analogue, it was possible to detect structure-dependent differences in activity. The *S*_P_ diastereomer α-thio-β,γ-methylene-ATP occurred to be the most promising candidate for improving skin wound healing and other pro-regeneration purposes. The *S*_P_-α-thio-β,γ-methylene-ATP was shown to induce purinergic signaling in human keratinocytes via interaction with the P2Y2 receptor thereby increasing the migration rate of these skin cells to close the wound area, which makes it a good candidate for skin regeneration applications.

Furthermore, we have shown herein for the first time not only the interaction of the P2Y2 receptor with diastereometrically pure α-thio-modified ATP analogues (both single- and double-modified), but also the stereoselectivity of this interaction and its consequences.

### Supplementary Information

Supplementary Information.

## Data Availability

All data generated or analyzed during this study are included in this published article and its supplementary information files.

## Abbreviations

ATCC: American Type Culture Collection
ATP: Adenosine-5′-triphosphate
DAPI: 4′,6-Diamidino-2-phenylindole
DMEM: Dulbecco’s modified Eagle’s medium
EMT: Epithelial–mesenchymal transition
HRMS: High-resolution mass spectra
MTD: Maximum Tolerated Dose
MTT reagent: 3-(4,5-Dimethyl-2-thiazolyl)-2,5-diphenyltetrazolium bromide
NDPKs: Ecto-nucleotide diphosphokinases
NPP1: Nucleotide pyrophosphatase/phosphodiesterase-1
NDP: Nucleoside diphosphate
NTPDase: Nucleoside triphosphate diphosphohydrolases
RMSD: Root mean-square deviation
RP-HPLC: Reverse phase high performance liquid chromatography
TEAB: Triethylammonium bicarbonate
TECs: Tracheal epithelial cells
TMPS: Thymidine-5′-O-monophosphorothioate
SASS: Self-assembled skin substitutes
UTP: Uridine-5′-triphosphate
