# Supplementary material for: Double-modified, thio and methylene ATP analogue facilitates wound healing in vitro and in vivo
Source: Sci Rep. 2024 Jun 7;14:13148. doi: 10.1038/s41598-024-63759-5 (PMC11161507; doi:10.1038/s41598-024-63759-5)
Supplement: Supplementary file 1 — Supplementary Information. [file 41598_2024_63759_MOESM1_ESM.docx]

**Supplementary Materials:**

**Double-modified, thio and methylene ATP analogue facilitates wound healing *in vitro* and *in vivo***

Roza Pawlowska^1^*, Ewa Radzikowska-Cieciura^1^, Sepideh Jafari^1,2^, Julia Fastyn^1,3^, Eliza Korkus^3^, Edyta Gendaszewska-Darmach^3^, Gangyin Zhao^4^, Ewa Snaar-Jagalska^4^, Arkadiusz Chworos^1^

*^1^Centre of Molecular and Macromolecular Studies, Polish Academy of Sciences, Sienkiewicza 112, 90-363 Lodz, Poland,*

*^2^BioMedChem Doctoral School of the University of Lodz and the Institutes of the Polish Academy of Sciences in Lodz, Lodz, Poland*

*^3^Institute of Molecular and Industrial Biotechnology, Faculty of Biotechnology and Food Sciences, Lodz University of Technology, Stefanowskiego 2/22, 90-537 Lodz, Poland*

*^4^ Institute of Biology, Leiden University, 2333 BE Leiden, The Netherlands*

**Corresponding author: roza.pawlowska@cbmm.lodz.pl*

***Figure S1.*** *The α-thio-ATP (****2****). Detailed data including results of ^1^H NMR, ^31^P NMR, HRMS ESI analysis and RP-HPLC profiles for* ***fast*** *and* ***slow*** *P-diastereoisomers of adenosine 5′-O-(P-α-thiotriphosphate) (****2a*** *and* ***2b****,* *respectively) are presented below. The original profiles of analysis were reported previously [31].*

Data for isomer **2a**: **^1^H NMR** (D_2_O, 200 MHz) δ: 8.48 (s, H-8, 1H), 8.02 (s, H-2, 1H), 5.94 (d, J= 3.8 Hz, H-1′, 1H), (H-2′ signal is hidden by the water signal at 4.64), 4.42 (m, H-3′, 1H), 4.16 (m, H-4′, 2xH-5’, 3H) ppm; **^31^P NMR** (D2O, 81 MHz) δ 43.40 (d, J = 27.7 Hz, Pα-S, 1P), -10.91 (d, J = 19.5 Pγ, 1P), -24.22 (dd, Jβγ = 20 Hz, Jαβ = 27.5Hz, Pβ, 1P) ppm; HRMS ESI (negative) m/z calculated for C10H12N5O11P3S− 521.9651, found 521.9657. The purity of compound was estimated using RP-HPLC analytical column: t*_R_* = 14.64 min (99% purity).

Data for isomer **2b**: **^1^H NMR** (D_2_O, 200 MHz) δ: 8.42 (s, H-8, 1H), 8.04 (s, H-2, 1H), 5.94 (d, J= 5.4 Hz, H-1′, 1H), (H-2′ signal is hidden by the water signal at 4.65), 4.44 (m, H-3′, 1H), 4.22 (m, H-4′, 2xH-5’, 3H) ppm; **^31^P NMR** (D2O, 81 MHz) δ 43.22 (d, J = 28 Hz, Pα-S, 1P), -10.64 (d, J = 19.5 Pγ, 1P), -24.19 (dd, Jβγ = 20 Hz, Jαβ= 27.5 Hz, Pβ, 1P) ppm; HRMS ESI (negative) m/z calculated for C10H11N5O11P3S− 521.9651, found 521.9656. The purity of compound was estimated using RP-HPLC analytical column: t*_R_* = 14.0 min (99% purity). The purity of compound was confirmed using RP-HPLC analytical column: t*_R_* = 16.15 min (96% purity).

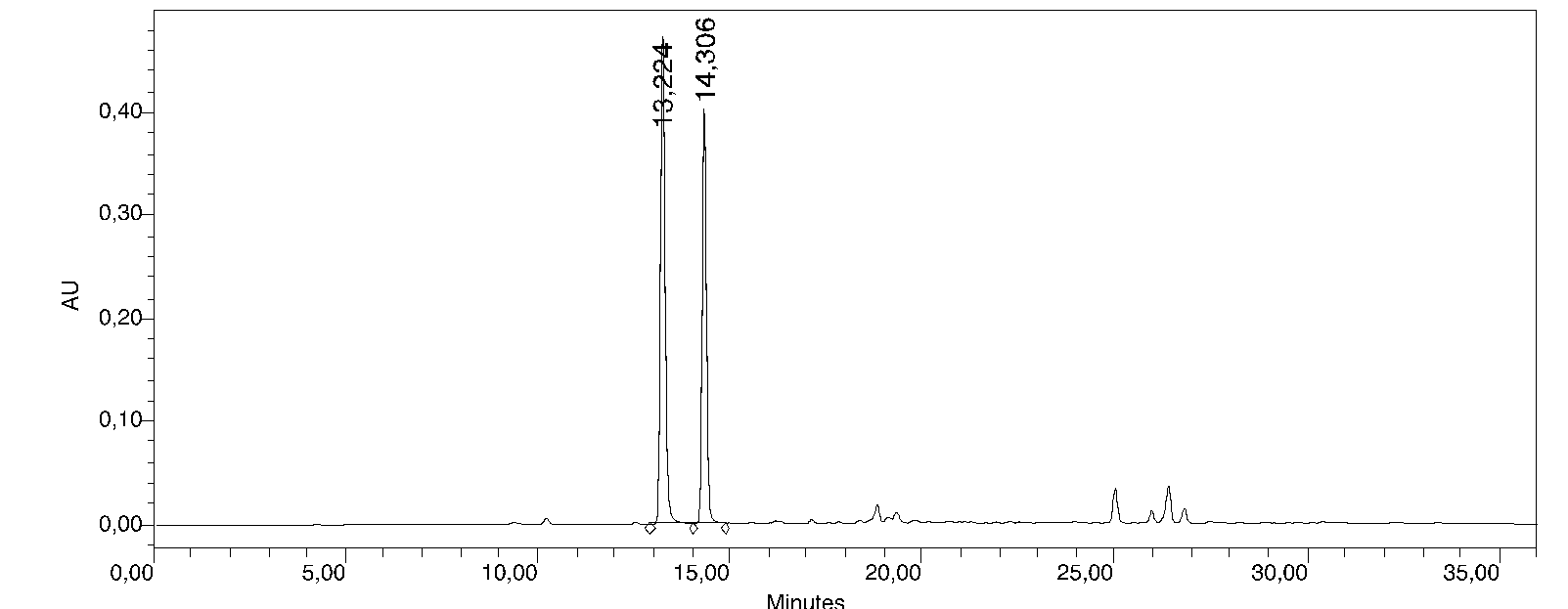


***Figure S2****. HPLC profile of adenosine 5*′*-O-(P-α-thio-β,γ-methylenetriphosphate) (****4****) after Sephadex purification.*


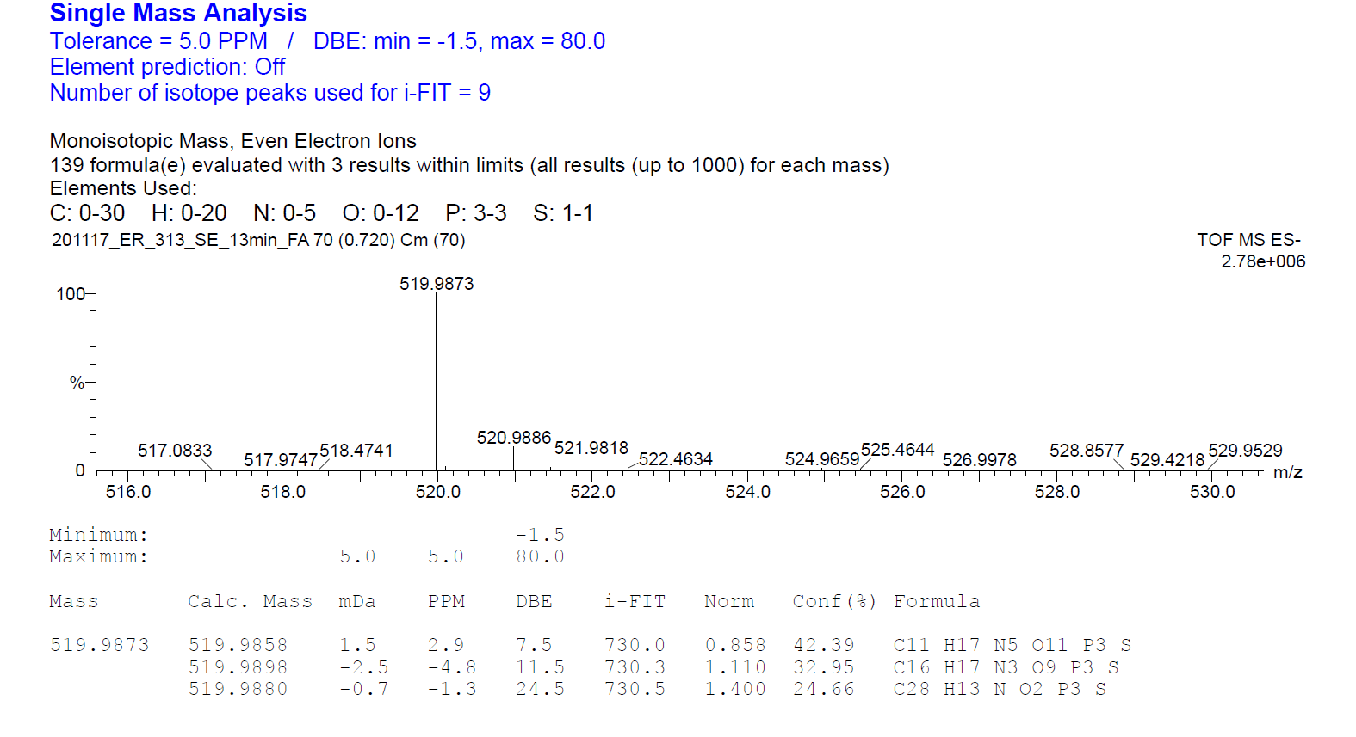


Data for isomer **4a**: ^1^H NMR (D2O, 200 MHz) δ 8.56 (s, H-8, 1H), 8.13 (s, H-2, 1H), 6.03 (d, J = 3.6 Hz, H-1′, 1H), 4.48 (m, H 2’ and H-3′, 2H), 4.31 (m, H-4′, 1H), 4.17 (m, H-5′, 2H), 2.21 (t, J = 20 Hz, CH2, 2H) ppm; ^31^P NMR (D2O, 81 MHz) δ 42.62 (d, J = 32 Hz, Pα-S, 1P), 14.44 (d, J = 8.4 Hz, Pγ, 1P), 8.38 (dd, J =αβ 32 Hz, Jαβ = 8.5 Hz, Pβ, 1P) ppm; HRMS ESI (negative) m/z calculated for C11H17N5O11P3S− 519.9858, found 519.9873. The purity of compound was confirmed using RP-HPLC analytical column: t*_R_* = 12.8 min (99% purity).

***Figure S3****. The mass analysis data for P-diastereoisomer* ***fast*** *of adenosine 5*′*-O-(P-α-thio-β,γ-methylenetriphosphate) (****4a****).*


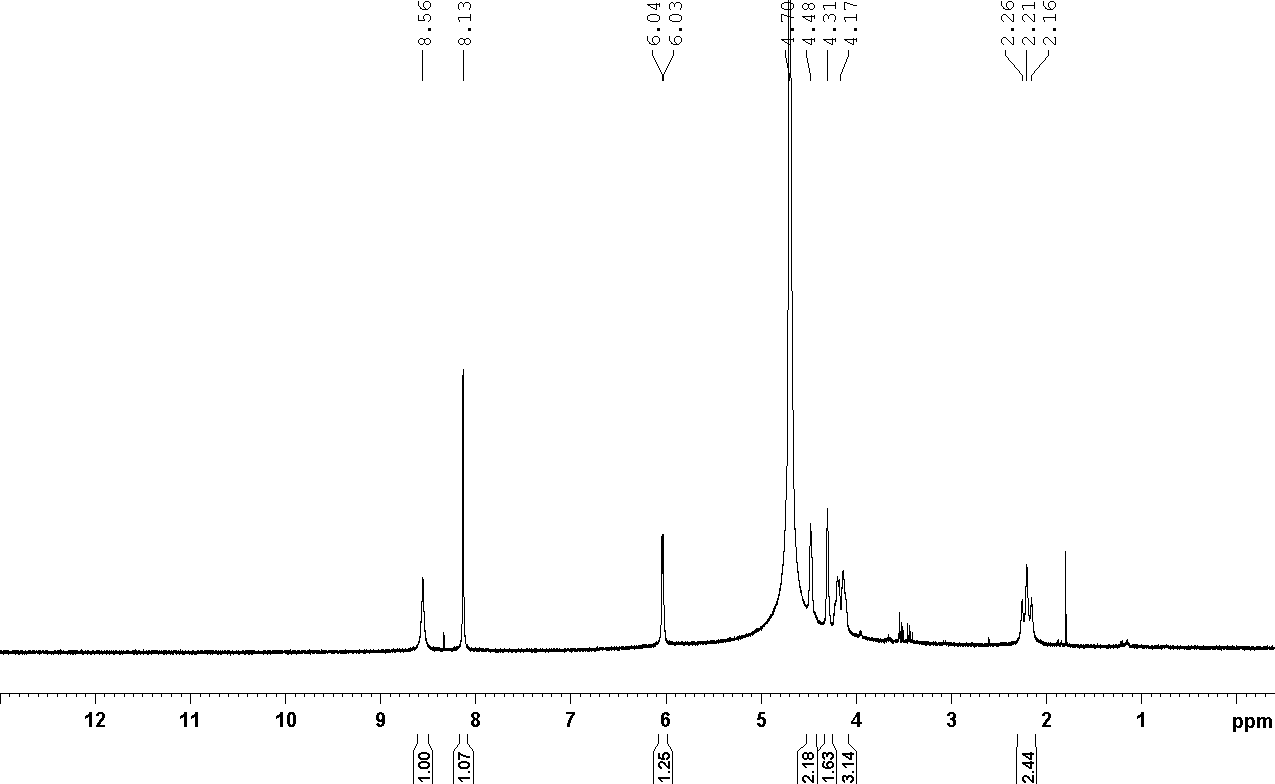


***Figure S4****. ^1^H NMR of P-diastereoisomer* ***fast*** *of adenosine 5*′*-O-(P-α-thio-β,γ-methylenetriphosphate) (****4a****) after HPLC purification.*


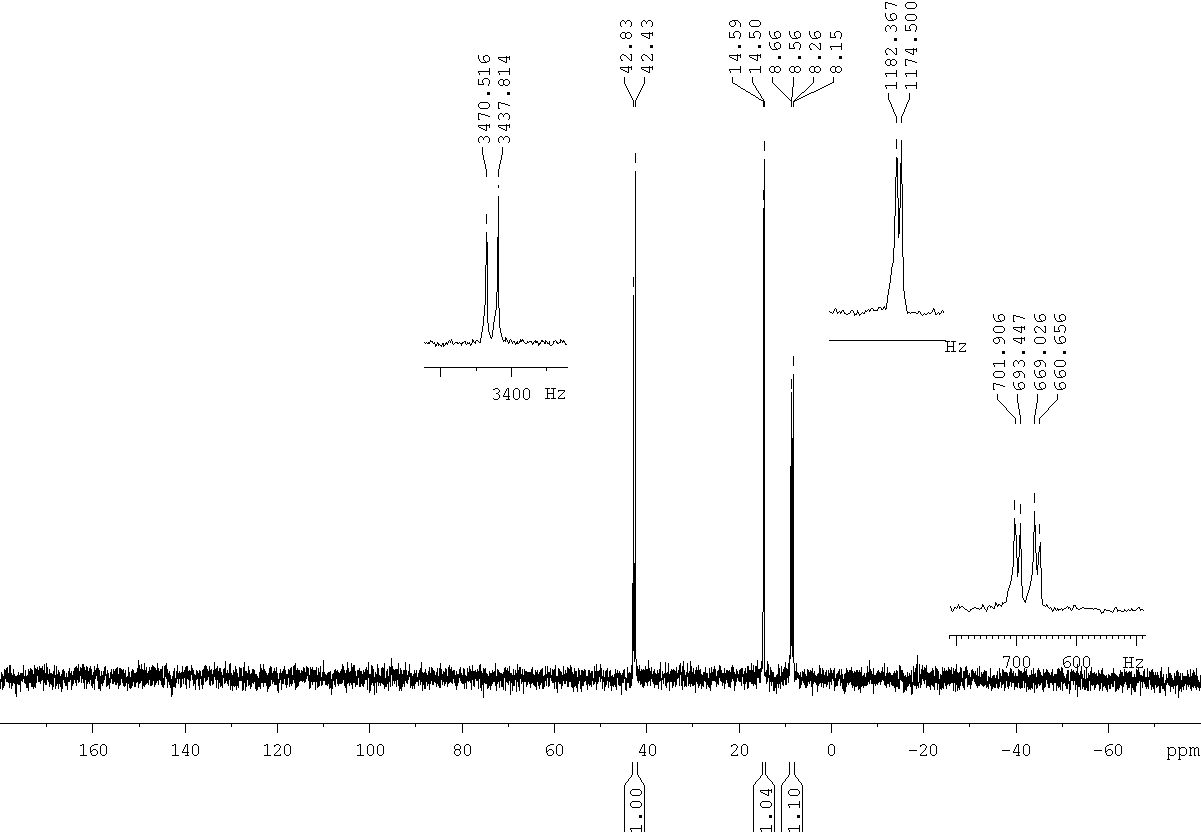


***Figure S5****. ^31^P NMR of P-diastereoisomer* ***fast*** *of adenosine 5*′*-O-(P-α-thio-β,γ-methylenetriphosphate) (****4a****) after HPLC purification.*


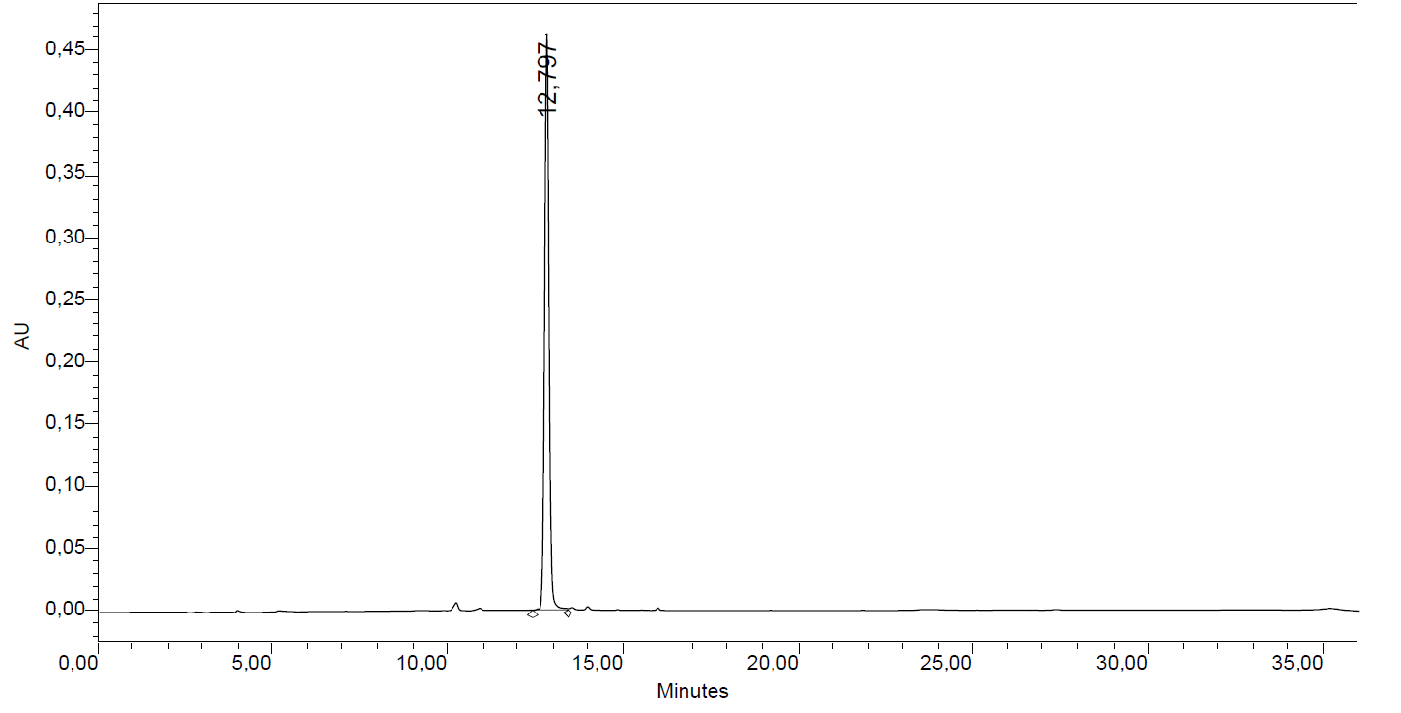


***Figure S6****. HPLC profile of P-diastereoisomer* ***fast*** *of adenosine 5*′*-O-(P-α-thio-β,γ-methylenetriphosphate) (****4a****) after HPLC purification.*

Data for isomer **4b**: 1H NMR (D2O, 200 MHz) δ 8.49 (s, H-8, 1H), 8.13 (s, H-2, 1H), 6.03 (d, J = 4.3 Hz, H-1′, 1H), 4.47 (m, H-2′*-* and H-3′, 2H), 4.31 (m, H-4′, 1H), 4.16 (m, H-5′, 2H), 2.25 (t, J = 20 Hz, CH2, 2H) ppm; ^31^ P NMR (D2O, 81 MHz) δ 42.45 (d, J = 33 Hz, Pα-S, 1P), 14.44 (d, J = 8.4 Hz, Pγ, 1P), 8.38 (dd, Jαβ = 33 Hz, Jαβ = 8.4 Hz, Pβ, 1P) ppm; HRMS ESI (negative) m/z calculated for C11H17N5O11P3S− 519.9858, found 519.9873. The purity of compound was confirmed using RP-HPLC analytical column: t*_R_* = 12.8 min (99% purity).

***Figure S7****. The mass analysis data for P-diastereoisomer* ***slow*** *of adenosine 5*′*-O-(P-α-thio-β,γ-methylenetriphosphate) (****4b****).*


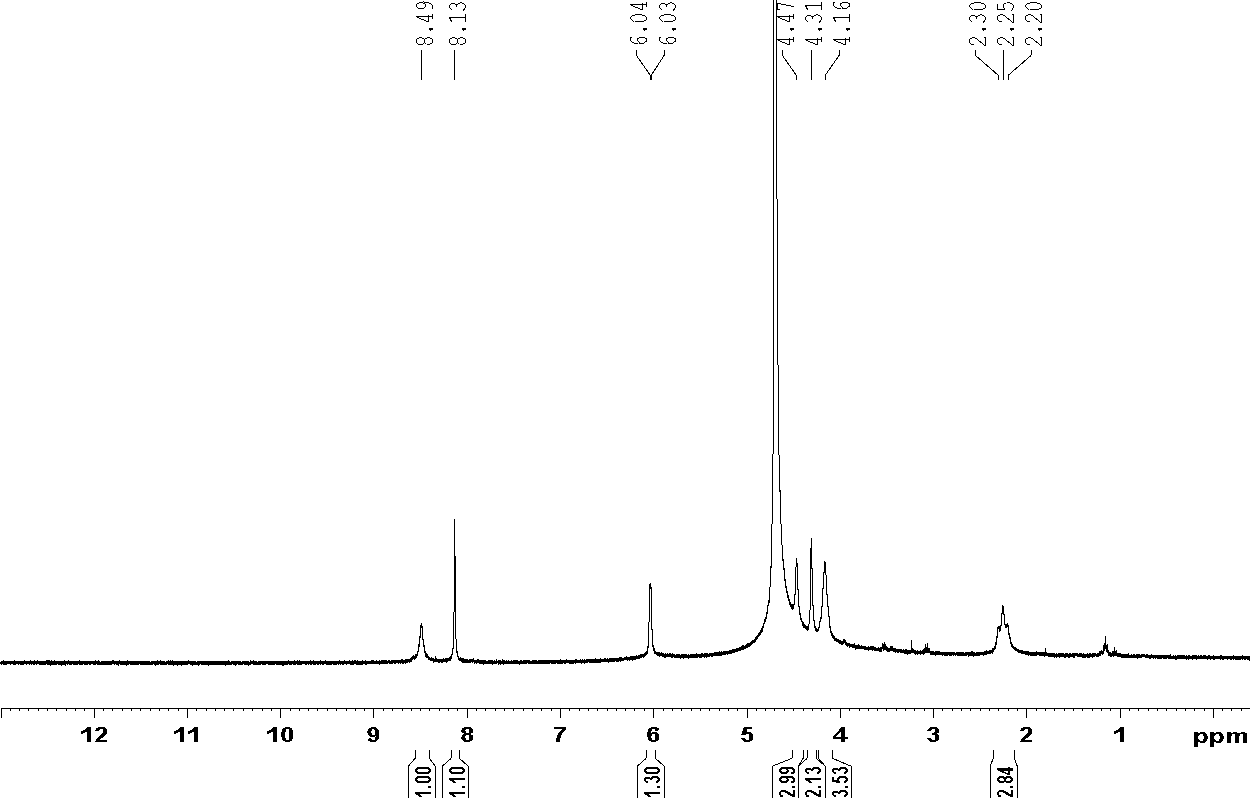


***Figure S8****. ^1^H NMR of P-diastereoisomer* ***slow*** *of adenosine 5*′*-O-(P-α-thio-β,γ-methylenetriphosphate) (****4b****) after HPLC purification.*


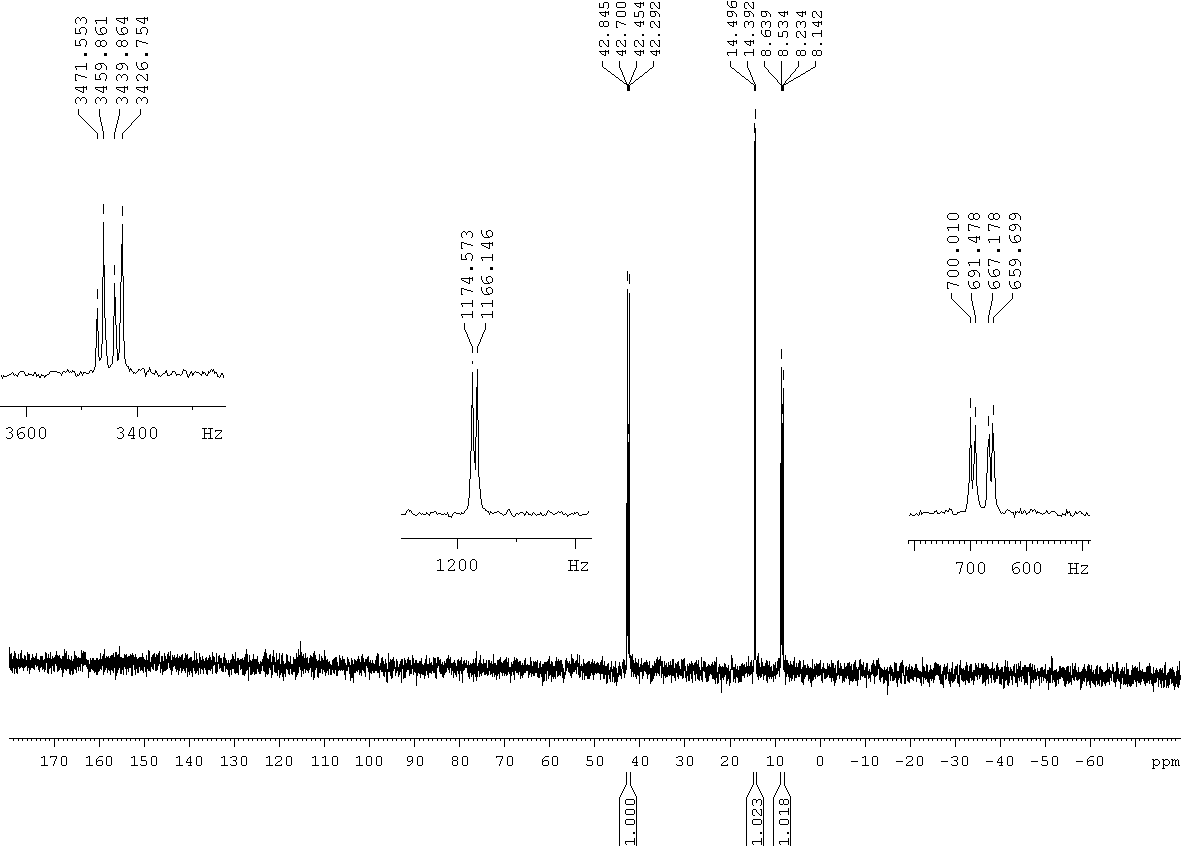


***Figure S9****. ^31^P NMR of P-diastereoisomer* ***slow*** *of adenosine 5*′*-O-(P-α-thio-β,γ-methylenetriphosphate) (****4b****) after HPLC purification.*


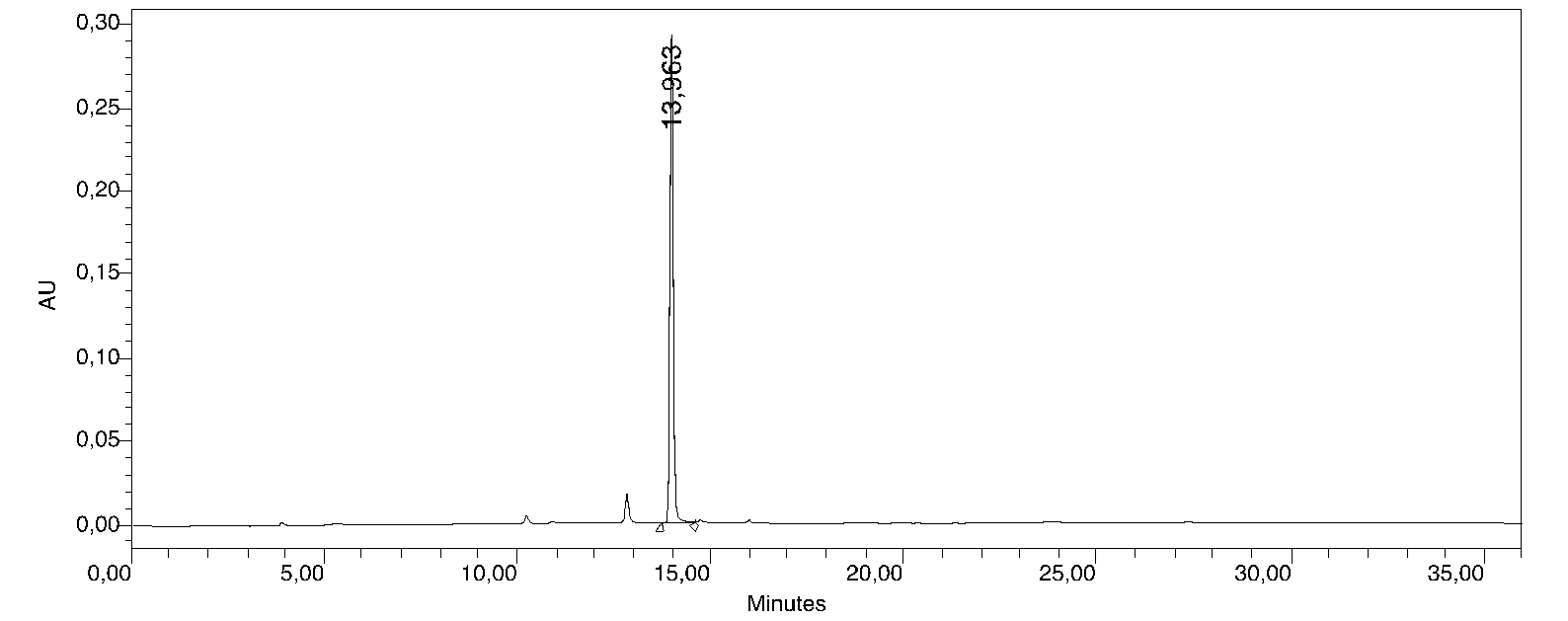


***Figure S10****. HPLC profile of P-diastereoisomer* ***slow*** *of adenosine 5*′*-O-(P-α-thio-β,γ-methylenetriphosphate) (****4b****).*


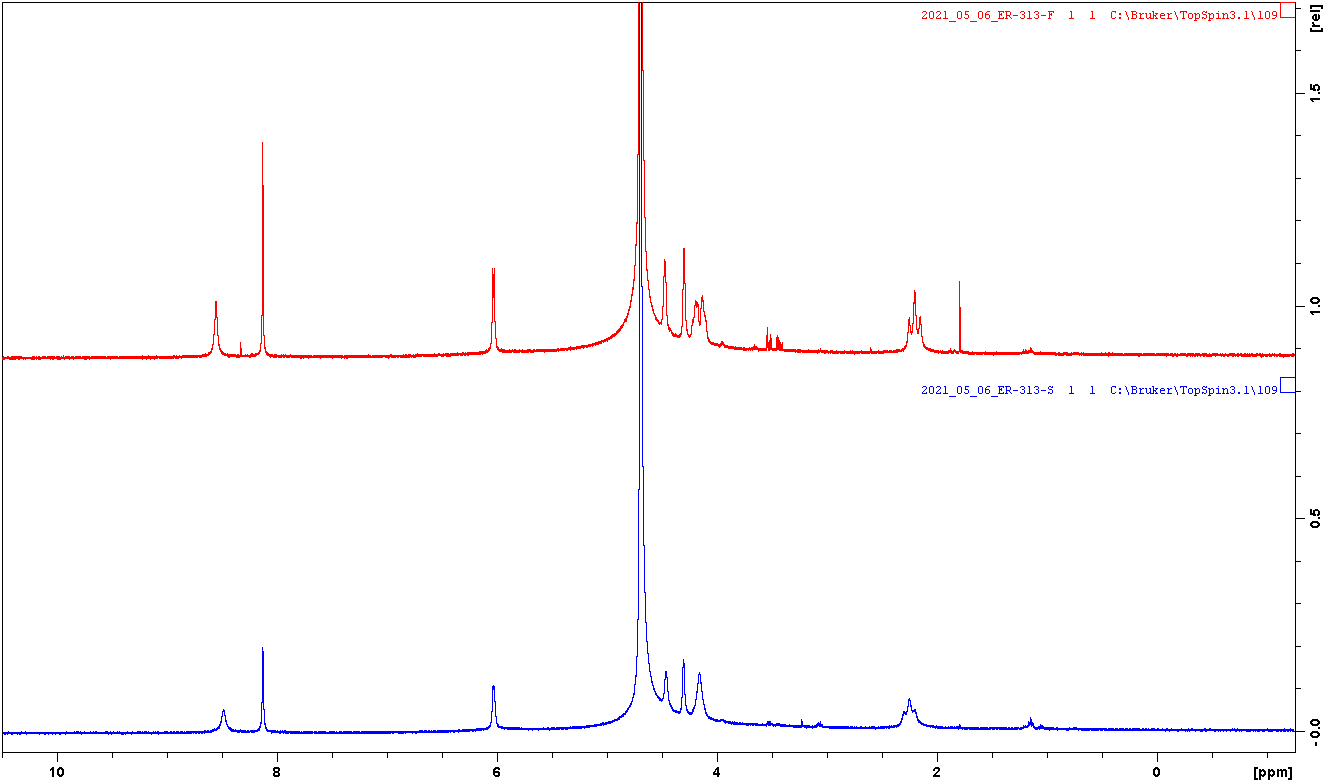


***Figure S11****. Overlay of ^1^H NMR spectra of P-isomers* ***fast*** *and* ***slow*** *of adenosine 5*′*-O-(P-α-thio-β,γ-methylenetriphosphate).*

***Table S1.*** *Determination of absolute configuration of the phosphorus atom in the α-thio-ATP analogues based on the NMR spectra and HPLC migration profiles. Data of NMR spectroscopy and retention time from HPLC analysis are taken from the profiles presented above and previously published by us [39]. The general structure of analyzed compounds with H numbering is provided below.*

| **α-thio-substituted nucleotide** | **^1^H NMR spectroscopy** | | | **Absolute configuration based on the NMR analysis** | **Retention time during HPLC (min)** | **Absolute configuration based on the HPLC analysis** |
| --- | --- | --- | --- | --- | --- | --- |
|  | **H-8** | **H-2** | **H-1’** |  |  |  |
| **2a** | 8.48 | 8.02 | 5.94  (J=3.8Hz) | *S*_P_ | 14.681 (fast) | *S*_P_ |
| **2b** | 8.42 | 8.04 | 5.95  (J=5.4Hz) | *R*_P_ | 16.145 (slow) | *R*_P_ |
| **4a** | 8.56 | 8.13 | 6.03  (J=3.6Hz) | *S*_P_ | 13.224 (fast) | *S*_P_ |
| **4b** | 8.49 | 8.13 | 6.03  (J=4.3Hz) | *R*_P_ | 14.306 (slow) | *R*_P_ |

*
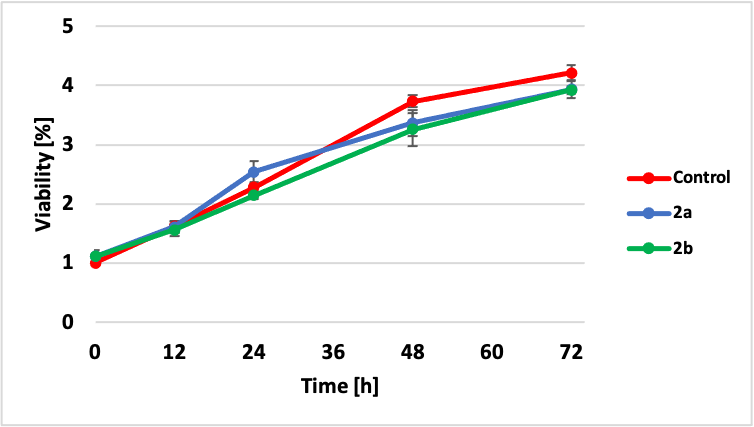
*

***Figure S12****. Viability of HaCaT cells after treatment with* ***2a*** *and* ***2b*** *compared to the Control (untreated cells). Data represent normalized average value of viability quantified by crystal violet staining ± SEM from at least 3 experiments performed in triplicate.*

*
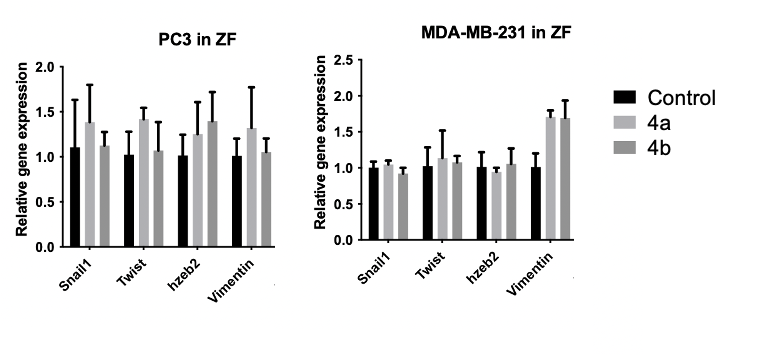
*

***Figure S13****. QPCR analysis of EMT markers isolated from the Zebrafish tails after PC3 and MDA-MB-231 cells injection into zebrafish xenografts and treatment with 20 μM α-thio-β,γ-methylene-ATP diastereomers (****4a*** *and* ***4b****).*

**

**

***Figure S14****. The mass spectrometry confirmation of the presence of* ***4a*** *in the fish culture water after Zebrafish experiment.*

******

******

***Figure S15****. The mass spectrometry confirmation of the presence of* ***4b*** *in the fish culture water after Zebrafish experiment.*

*
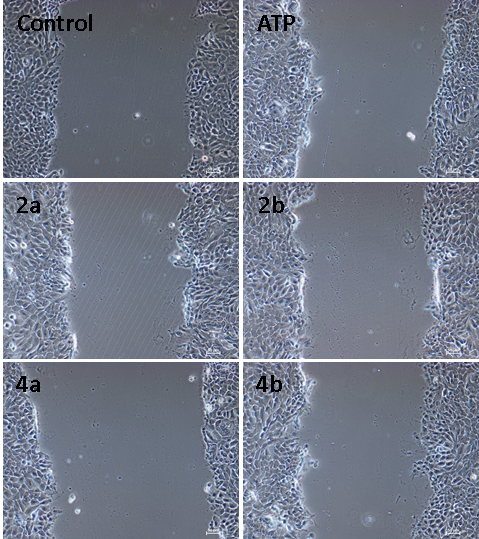
*

***Figure S16****. The microscopy images of the initial scratch area in the wound healing experiments using human keratinocytes HaCaT.*

*
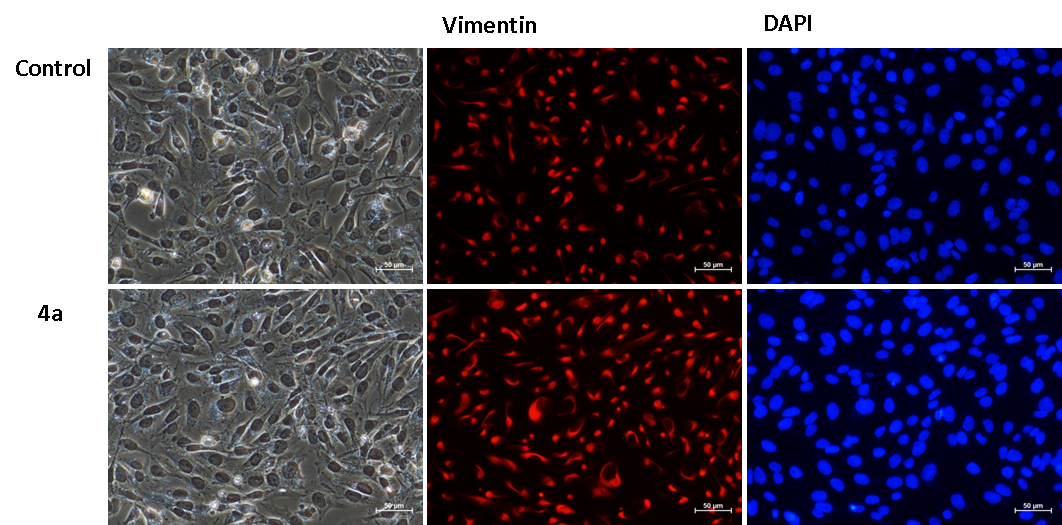
*

***Figure S17****. The immunocytochemical (ICC) visualization of the vimentin in the MDA-MB-231 cells after treatment with S*_P_ *diastereomer of α-thio-β,γ-methylene-ATP (****4a****) and in control sample (untreated cells). Scale bars 50 μm.*

*
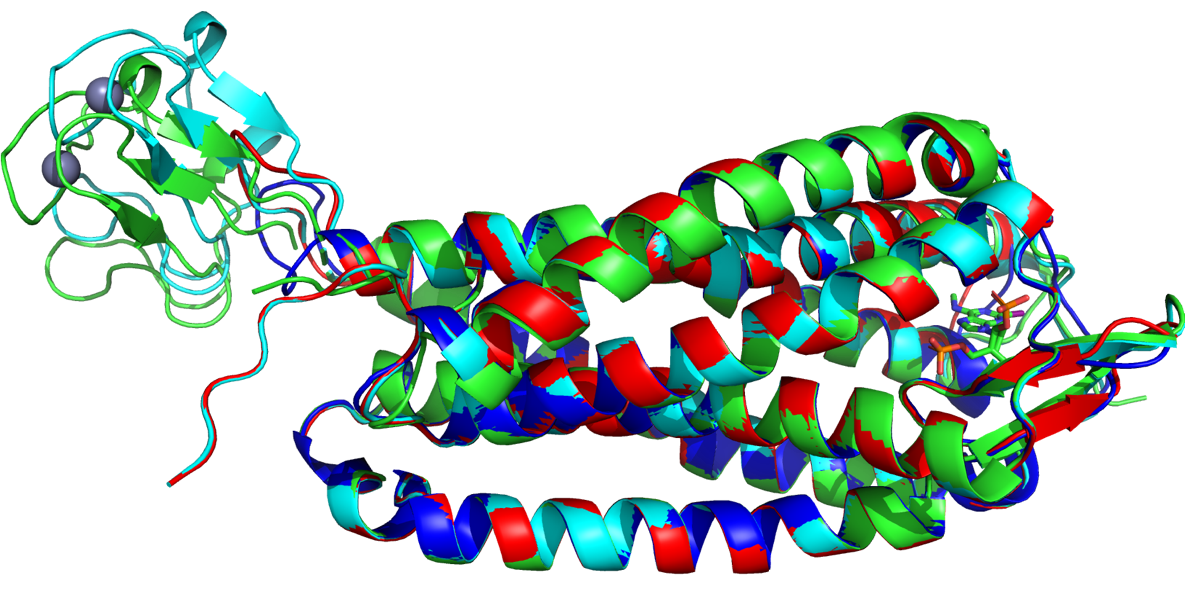
*
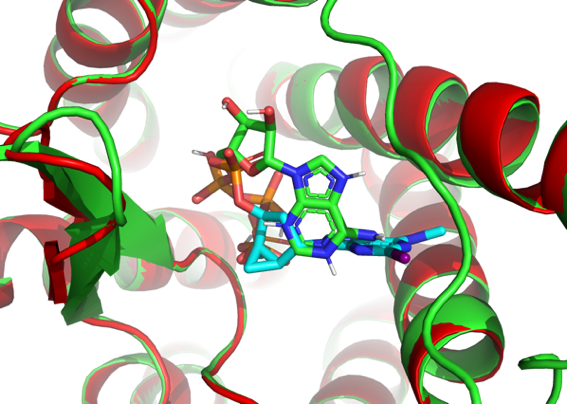


***Figure S18****. The structure comparison between (left): P2Y1 receptor (crystal structure PDB: 4XNW) chain A (green), chain C (cyan), a homology model of P2Y2 receptor by Rafehi et. al. [47] (dark blue) and this study homology model of P2Y2 receptor (red), (right): the location of the active center in the human P2Y1 receptor (PDB: 4XNW) with 2-iodo-6-(methyl) adenine derivative as it was crystalized (C-blue) and active center in the homology model of P2Y2 receptor with ATP (C-green).*

*
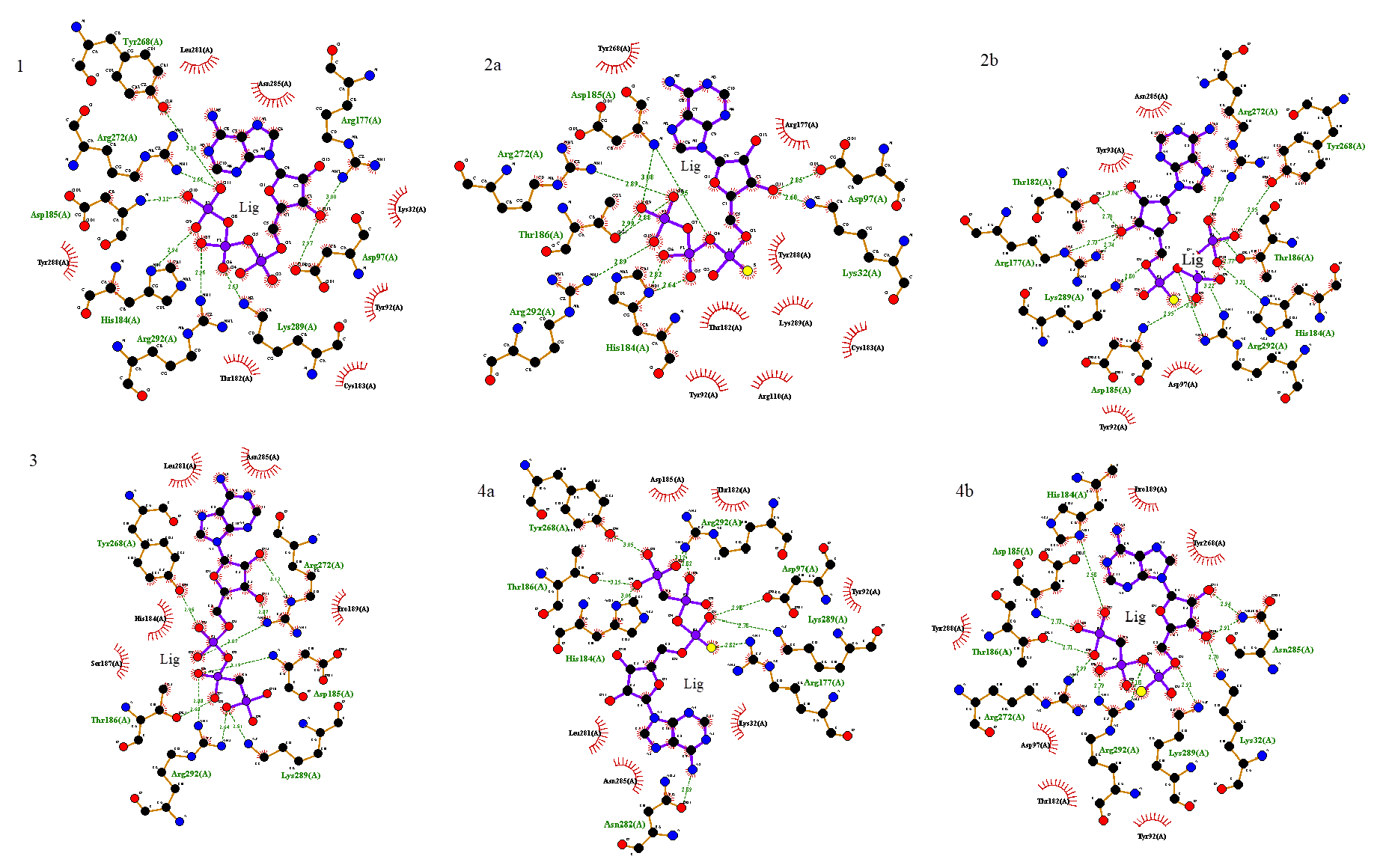
*

***Figure S19****. The LigPlot graph presents the 2D plot of P2Y2 receptor-ligand interactions with ATP (****1****) and ATP analogues (****2a-4b****).*
